# Phylotranscriptomic insights into a Mesoproterozoic–Neoproterozoic origin and early radiation of green seaweeds (Ulvophyceae)

**DOI:** 10.1038/s41467-022-29282-9

**Published:** 2022-03-22

**Authors:** Zheng Hou, Xiaoya Ma, Xuan Shi, Xi Li, Lingxiao Yang, Shuhai Xiao, Olivier De Clerck, Frederik Leliaert, Bojian Zhong

**Affiliations:** 1 College of Life Sciences, Nanjing Normal University, Nanjing, China; 2 Department of Geosciences and Global Change Center, Virginia Tech, Blacksburg, VA USA; 3 Phycology Research Group and Center for Molecular Phylogenetics and Evolution, Ghent University, Ghent, Belgium; 4 Meise Botanic Garden, Meise, Belgium

**Keywords:** Phylogenetics, Plant evolution

## Abstract

The Ulvophyceae, a major group of green algae, is of particular evolutionary interest because of its remarkable morphological and ecological diversity. Its phylogenetic relationships and diversification timeline, however, are still not fully resolved. In this study, using an extensive nuclear gene dataset, we apply coalescent- and concatenation-based approaches to reconstruct the phylogeny of the Ulvophyceae and to explore the sources of conflict in previous phylogenomic studies. The Ulvophyceae is recovered as a paraphyletic group, with the Bryopsidales being a sister group to the Chlorophyceae, and the remaining taxa forming a clade (Ulvophyceae *sensu stricto*). Molecular clock analyses with different calibration strategies emphasize the large impact of fossil calibrations, and indicate a Meso-Neoproterozoic origin of the Ulvophyceae (*sensu stricto*), earlier than previous estimates. The results imply that ulvophyceans may have had a profound influence on oceanic redox structures and global biogeochemical cycles at the Mesoproterozoic-Neoproterozoic transition.

## Abstract (web summary)

“Ulvophyceae is a remarkably morphologically and ecologically diverse clade of green algae. Here, the authors reconstruct the Ulvophyceae phylogeny, showing that these algae originated earlier than expected and may have influenced biogeochemical cycles at the Mesoproterozoic-Neoproterozoic transition.”

## Introduction

The Chlorophyta is a large and ancient clade of green plants (Viridiplantae), including morphologically diverse green algae living in a wide range of habitats. The Ulvophyceae, one of the main classes of the Chlorophyta, displays an extraordinary diversity, ranging from unicellular or multicellular organisms to giant-celled siphonous or siphonocladous thalli^1,2^. Most species are marine macroalgae (green seaweeds), which are important primary producers in coastal ecosystems, and have important ecological functions as ecosystem engineers^3^. A smaller diversity is found in freshwater and terrestrial habitats^3,4^. Rapid growth of some green seaweeds (e.g., *Ulva*, *Cladophora*) under high temperature and nutrient conditions can lead to green algal blooms in coastal waters, also known as “green tides”, with negative economic and ecological impacts. Ulvophyceans are also economically important. For example, species in the genera *Ulva*, *Caulerpa*, and *Cladophora* produce sustainable biomass material for the food, aquaculture and biofuel industries, as well as medicine and pharmacology^3,5^. A reliable phylogenetic framework and an evolutionary timeline of the Ulvophyceae are urgently needed to improve our understanding of the evolutionary innovations of these green seaweeds, such as their unique cytomorphological features^6^, and carbon concentrating mechanisms which contribute to the high growth rates of some ulvophyceans^7^.

**Fig. 1:**
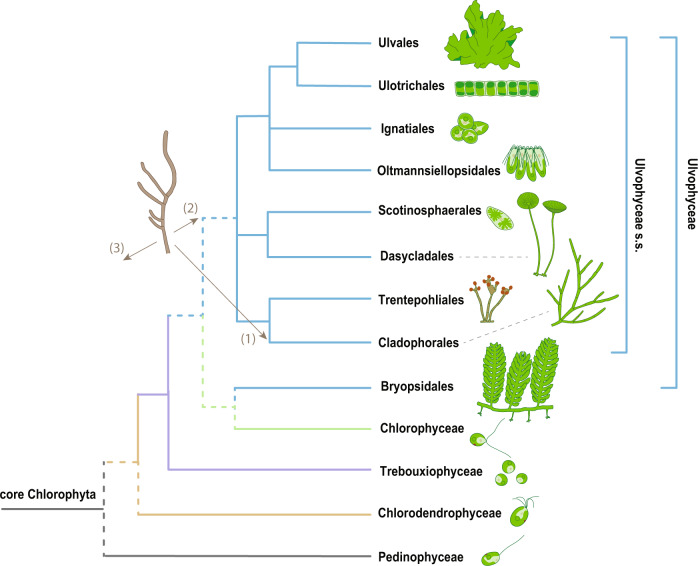
Current knowledge of the phylogenetic relationships among the main lineages of the core Chlorophyta based on nuclear phylogenomic studies^17,18,20^. Uncertain phylogenetic relationships are indicated by polytomies or dashed lines. The three different interpretations of the *Proterocladus antiquus* fossil, corresponding to the three strategies used in our dating analyses, are indicated with arrows: (1) *Proterocladus* as a total-group cladophoralean, (2) *Proterocladus* as a total-group ulvophycean, and (3) uncertain phylogenetic position (within or outside the green algae).

The phylogenetic relationships among the main lineages of the Ulvophyceae have been difficult to resolve (Fig. 1). Nuclear ribosomal and chloroplast multigene data produced inconsistent and contradictory results^2^. Furthermore, the monophyly of the Ulvophyceae has been questioned based on the lack of synapomorphic characters (including ultrastructural features of the flagellate reproductive cells and modes of nuclear division) distinguishing them from the related Chlorophyceae and Trebouxiophyceae^8,9^ (Supplementary Note 1 and Supplementary Table 1). Although these ultrastructural features were believed to accurately reflect phylogenetic relationships because of their involvement in fundamental processes of cell replication and cell motility, analyses of these data have not been able to resolve the phylogenetic relationships among the Ulvophyceae, Chlorophyceae, and Trebouxiophyceae, or among the orders of the Ulvophyceae^10^. The advent of molecular methods provided new opportunities for reconstructing the phylogeny of the green algae^2^. Early phylogenies based on ribosomal DNA have recovered the Ulvophyceae as a monophyletic group but with weak support^11,12^. A 10-gene phylogeny (eight nuclear and two plastid genes) recovered the Ulvophyceae as a well-supported clade for the first time, and elucidated the relationships of the main lineages^6^. More recently, however, chloroplast phylogenomic analyses have rejected the monophyly of the Ulvophyceae, and instead indicated that several ulvophycean lineages were related to other core chlorophytan clades^13–16^. Phylotranscriptomic analyses partly confirmed these chloroplast phylogenomic studies, supporting a non-monophyletic Ulvophyceae with the Bryopsidales being sister to the Chlorophyceae, or indicating a hard polytomy among Chlorophyceae, Bryopsidales, and the remaining Ulvophyceae^17–20^, in which the relative branching order of these taxa cannot be resolved with confidence. However, these genome-scale analyses have suffered from relatively low taxon sampling (e.g., in the orders Dasycladales and Cladophorales), and factors such as long-branch attraction (LBA) artifact and ancient rapid radiation likely confounded phylogenetic relationships^12,13,21^. Therefore, richer taxon sampling, genome-wide nuclear datasets, and application of more plausible evolutionary models are expected to further enhance our knowledge of phylogenetic relationships within the Ulvophyceae.

Estimating a timeframe for diversification of the green algae has been an equally difficult task given the absence of biomineralization in most groups of green algae, resulting in poor fossil records. In addition, the phylogenetic interpretation of alleged green algal fossils, particularly unicellular fossils with simple morphologies, is a significant challenge because of known morphological convergence in the evolution of protists and algae^22,23^. Thus, the taxonomic interpretation of Precambrian green algal fossils has been heavily debated, limiting their value as fossil calibration points in molecular clock analyses. Only a few orders of Ulvophyceae that include species with calcified thalli (e.g., Bryopsidales and Dasycladales) have a relatively rich fossil record, but these fossils are generally younger, occurring from the Paleozoic onward^24–26^. A notable Precambrian fossil genus is *Proterocladus*, characterized by branched filamentous thalli, and first described from Neoproterozoic deposits of ca. 750 mya (refs. ^24,27^). Recently, a new species, *P. antiquus*, has been described from one-billion-year-old deposits^28^, prompting further interest in the phylogenetic and molecular clock analyses of the Ulvophyceae. Based on the apparent coenocytic thallus architecture and its resemblance with extant *Cladophora* and *Cladophoropsis* species, *Proterocladus* has been attributed to the Cladophorales, although its taxonomic placement remains contentious^18,29^. Thus, it is appropriate to more thoroughly evaluate *Proterocladus* in a phylogenetic context to better constrain the evolution of ulvophyceans. A more accurate phylogeny and evolutionary timeline of chlorophytes and ulvophyceans would in turn improve our understanding of the potential impact of green algae on the Mesoproterozoic–Neoproterozoic transition that bridges the “boring billion” (an apparently quiescent period in the global carbon cycle between 1600 and 1000 Ma) and the “freezing millions” (three glaciation events at about 717–662, 654–635, and 580 Ma)^30^.

In this study, we carried out an intensive nuclear gene sampling including 11 new ulvophycean transcriptomes, and constructed a multigene dataset containing 884 genes and 69 species (including 43 ulvophyceans). We applied coalescent- and concatenation-based phylogenetic approaches, and took the heterogeneity of genes, sites and lineages into account in the model selection. We resolved the backbone relationships of the Ulvophyceae, and evaluated the diversification time estimates through three fossil assignment strategies to account for the alternative interpretations of *P. antiquus*. Notably, our results suggest important connections between the early evolution of green seaweeds and significant changes in the Earth system during and immediately after the Mesoproterozoic–Neoproterozoic transition.

## Results

### Phylogenomic analyses of the core Chlorophyta

**Fig. 2:**
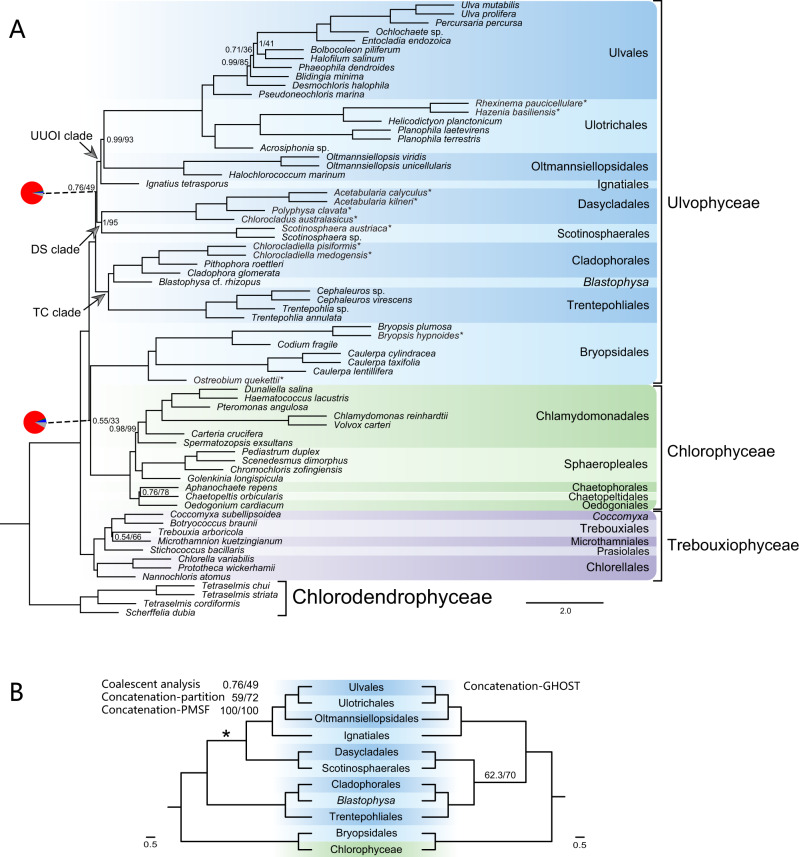
Phylogenetic relationships of Ulvophyceae inferred from 884 nuclear genes. **A** Species tree based on the multispecies coalescent model. Support for each node is provided by local posterior probability and multi-locus bootstrapping (PP/MLBS). Nodes without a value indicate full support. The pie charts at two focal nodes of Ulvophyceae present the proportion of gene tree concordance and conflict. Pie chart color coding: blue, fraction of gene trees that are concordant with the species tree; green, fraction of gene trees supporting the second most common conflicting topology; red, fraction of gene trees supporting all other alternative conflicting partitions; gray, fraction of gene trees with <50% bootstrap support at that node. The newly sampled species are marked with a symbol (*). Note that *Desmochloris* has been placed in the Chlorocystidales^105^, and *Pseudoneochloris* has been recently placed in a new order, Sykidiales^106^. **B** Topological comparison among coalescent- and concatenation-based analyses. Support for the conflicting branches is provided by PP/MLBS (in coalescent-based analysis) and SH-aLRT/ultrafast bootstrap (in concatenation-based analyses). The asterisk indicates support in the three analyses on the left. The source data of the pie charts in (**A**) are provided in the Source Data file.

Our dataset included 884 nuclear genes from 69 species of Ulvophyceae, Chlorophyceae and Trebouxiophyceae, and four Chlorodendrophyceae species as outgroup. We applied coalescent- and concatenation-based approaches to reconstruct phylogenetic relationships. All the phylogenetic trees were largely congruent and most relationships among major lineages of core Chlorophyta were well-supported. Trebouxiophyceae and Chlorophyceae were inferred as monophyletic groups with full support, and the former was sister to a clade containing the Ulvophyceae and Chlorophyceae (Fig. 2A, Supplementary Figs. 1–3). The Ulvophyceae, however, was recovered as paraphyletic. Bryopsidales and Chlorophyceae were recovered as a clade in all analyses, with full support in the concatenation-based analyses, and weak support in the coalescent-based analysis (0.55/33, PP/MLBS, Fig. 2A). The remaining orders of Ulvophyceae (hereafter Ulvophyceae *sensu stricto* or s.s.) were grouped in three subclades: (1) the UUOI clade, which successively placed Ignatiales, Oltmannsiellopsidales, and Ulotrichales as sister to Ulvales, (2) the DS clade consisting of Dasycladales and Scotinosphaerales, and (3) the TC clade including the Trentepohliales and Cladophorales plus *Blastophysa*. Within each of these subclades, relationships were concordant among the different analyses (Fig. 2A, Supplementary Figs. 1–3). Incongruence was found in the position of the DS clade (Fig. 2B). The DS and UUOI clades were sister in the coalescent-based analysis, as well as in the concatenation-based analyses using partition strategy and posterior mean site frequency (PMSF) model (Fig. 2A, Supplementary Figs. 1, 2), while the concatenation-based analysis using a heterotachy model supported a sister relationship between the DS and TC clades (Supplementary Fig. 3). The phylogenetic conflict in the concatenation-based analyses implied that the relationships among these deep lineages of the Ulvophyceae (i.e., DS and TC clades) were sensitive to model selection.

### Gene tree discordance and coalescent simulations

**Fig. 3:**
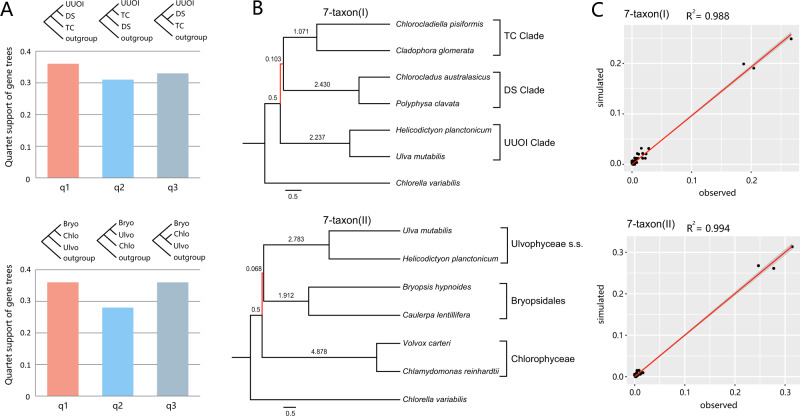
Gene tree discordance and coalescent simulations. **A** The frequency of three topologies (q1–q3) around two short internal branches of ASTRAL species tree in the 69-taxon datasets. Each short internal branch with four neighboring branches leads to three possible topologies. **B** Phylogenetic relationships of 7-taxon(I) and 7-taxon(II) datasets based on the multispecies coalescent model. Red branches are the short branches. **C** Comparison of simulated frequencies of gene tree topologies with the corresponding observed frequencies. Bryo Bryopsidales, Chlo Chlorophyceae, Ulvo Ulvophyceae s.s.

In the coalescent-based analysis, the UUOI and DS clades as well as the Bryopsidales–Chlorophyceae relationship were separated by extremely short and weakly supported branches (Fig. 2A). In addition, conflicts between gene trees and the coalescent species tree were particularly situated in these short branches (Supplementary Fig. 4). The proportions of conflicting bipartitions for the UUOI-DS node, and the Bryopsidales–Chlorophyceae nodes were 92.76% and 87.10% respectively, and the concordant proportions were only 2.15% and 4.75%, respectively (pie charts in Fig. 2A). We also detected gene tree discordance using quartet supports among three topologies (q1–q3) of the focal internal branch in the coalescent-based analysis. Of the 884 gene trees, 36% supported the main topology ((DS, UUOI), TC), 31% supported a first alternative topology ((UUOI, TC), DS), and the remaining 33% supported a second alternative topology ((DS, TC), UUOI) (Fig. 3A). Similarly, 36% of 884 gene trees supported Bryopsidales as sister to Chlorophyceae, 28% supported Bryopsidales as sister to the Ulvophyceae s.s., and the other 36% supported Chlorophyceae as sister to the Ulvophyceae s.s. (Fig. 3A). The short branches and similar topological frequencies (q1–q3) of gene trees are suggestive of incomplete lineage sorting (ILS) in the deep lineages of Ulvophyceae.

To further test whether the short branches and gene tree discordance were caused by ILS, we assembled two small datasets [7-taxon(I) and 7-taxon(II)] and performed coalescent simulations. We separately simulated 100,000 gene trees from 7-taxon(I) and 7-taxon(II) species trees under the multispecies coalescent model (Fig. 3B), and compared the topological frequencies of simulated gene trees with observed gene trees. There was a significant correlation between the simulated and observed topologies (Pearson’s correlation coefficient = 0.994, *p* < 0.01 for 7-taxon(I) dataset; Pearson’s correlation coefficient = 0.997, *p* < 0.01 for 7-taxon(II) dataset, Fig. 3C), indicating that ILS could well explain the topological conflicts among the main lineages of Ulvophyceae. The multispecies coalescent model can accommodate gene tree heterogeneity and has been proven to be able to produce more accurate species trees in the presence of ILS (refs. ^31,32^). Therefore, we used the phylogenetic relationship of the Ulvophyceae reconstructed by the multispecies coalescent model as a framework for subsequent analyses.

### Time-calibrated phylogeny of the Ulvophyceae

**Fig. 4:**
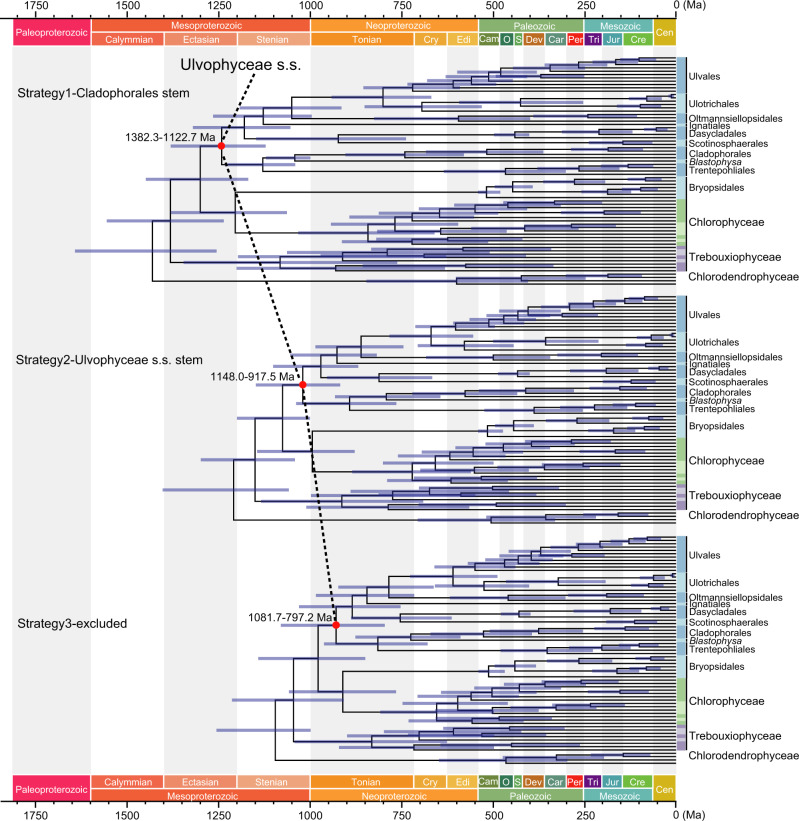
Comparison of divergence time estimates from three fossil assignment strategies. The differences between the three calibration strategies refer to the placement of *Proterocladus antiquus* (strategy 1: *P. antiquus* to stem-group Cladophorales; strategy 2: *P. antiquus* to stem-group Ulvophyceae s.s.; strategy 3: exclusion of *P. antiquus*). Node ages are plotted at the posterior means, with horizontal bars representing 95% credibility intervals.

Our phylogenomic results provided a phylogenetic framework for estimating divergence times in ulvophycean evolutionary history. Time-calibrated phylogenetic analyses were performed using a Bayesian relaxed clock model in MCMCTree, based on the coalescent species tree and 200 most clock-like genes. We compared the posterior distributions to the effective prior distributions for the different calibrated nodes (Supplementary Fig. 5). The effective priors were generated without molecular data, and posteriors were obtained using data. Most fossil-calibrated nodes demonstrated shifts between posterior and effective prior, confirming that posterior time estimates are not simply dependent on priors, but also on the contribution of molecular data. Our results of three calibration strategies indicated that divergence times of Ulvophyceae s.s. was sensitive to the placement of the one-billion-year-old fossil *Proterocladus antiquus* (Fig. 4, Supplementary Figs. 6–8). In strategy 1, assignment of *P. antiquus* to the stem-group Cladophorales resulted in an estimated origin of the Ulvophyceae s.s. in the Mesoproterozoic Era (95% CI = 1382.3–1122.7 Ma). Analyses where *P. antiquus* was assigned to the stem-group Ulvophyceae s.s. (strategy 2) or was excluded (strategy 3), resulted in younger node age estimates of the Ulvophyceae s.s. (strategy 2: 95% CI = 1148.0–917.5 Ma, strategy 3: 95% CI = 1081.7–797.2 Ma). The younger nodes, such as those of the ulvophycean orders, yielded similar time estimates in all three calibration strategies. Our analyses thus indicate that the divergence of the Ulvophyceae s.s. occurred in the late Mesoproterozoic to early Neoproterozoic; subsequently, various ulvophycean lineages (e.g., Dasycladales, Trentepohliales) diversified in the Neoproterozoic and Paleozoic.

## Discussion

### Phylogenetic relationships and morphological evolution of Ulvophyceae

The recent explosive growth of omics data and application of more realistic evolutionary models are rewriting our understanding of the relationships and temporal patterns of diversification of many taxa. Ulvophyceae is not an exception to this. In agreement with recent studies^17–20^, our phylogenomic analyses indicate that the Ulvophyceae is non-monophyletic, with the Bryopsidales and the Chlorophyceae forming a clade, which is sister to the Ulvophyceae s.s. (Fig. 2, Supplementary Figs. 1–3). Increased taxon sampling largely confirmed the relationships within the Ulvophyceae s.s., supporting three main clades (UUOI, DS, and TC), and enabled resolving the phylogenetic relationships within these clades with higher confidence. The relationships within the UUOI clade have been contentious^11,12,18,33^. Some studies placed the Oltmannsiellopsidales as a sister clade to the clade containing Ignatiales, Ulotrichales, and Ulvales, but these relationships were either based on a limited number of genes (e.g., small subunit rDNA and/or *rbc*L sequences, refs. ^11,12^), or received moderate to poor support^17,18^. Our analyses are consistent with chloroplast phylogenomic analyses, which also showed a relatively robust support for the relationships among these four orders, with the Ignatiales branching off first^33^. Conversely, some nuclear phylogenomic analyses resolved the Oltmannsiellopsidales as a sister clade to the DS clade^20^.

This study confirms the sister relationships of the Dasycladales and Scotinosphaerales, as well as the Trentepohliales and Cladophorales-*Blastophysa* of previous nuclear phylogenomic studies^18,20^. Phylogenetic analyses based on one or a few genes, as well as chloroplast genomes have largely failed to resolve the relationships among these orders^6,11,12,34,35^. The contentious relationships of these orders were likely caused by insufficient taxon sampling, for instance the notable absence of Scotinosphaerales^11^ or Cladophorales^15^ in chloroplast-based analyses. In addition, LBA appearing in several phylogenetic analyses of the Ulvophyceae also likely biased the topology^12,13,21,34^. In the present study, relationships within the DS and TC clades were well resolved, and we greatly alleviated long branches in the Trentepohliales and Cladophorales by denser taxon sampling, and by employing site-heterogeneous and heterotachy models. In agreement with previous phylogenomic studies^17,18,20^, most of our analyses did not support a sister relationship between TC and DS clades.

The inferred phylogenetic relationships provide a solid framework to further our understanding of cytomorphological evolution in the green seaweeds. Ancestral state reconstruction confirmed that multicellularity, as well as the siphonous thallus architecture evolved independently and repeatedly in various lineages of the Ulvophyceae, likely from unicellular uninucleate ancestors^6,17,18^ (Supplementary Figs. 9, 10). Our phylogeny also provides a framework for re-evaluating earlier hypotheses of the evolution of cytological features that were believed to be phylogenetic informative at higher taxonomic level, such as the ultrastructure of the flagellar root system and the processes of mitosis and cell division (Supplementary Note 1 and Supplementary Table 1). Some ultrastructural features, such as the processes of mitosis and cytokinesis, may be less conserved than previously thought, as for example illustrated by the fundamentally different processes in the sister clades Cladophorales and Trentepohliales. However, information on ultrastructural features is largely lacking for the smaller ulvophycean lineages, hampering a complete picture of ultrastructural evolution in the group.

Given the conflicting relationships among the UUOI, DS, and TC clades, combined with the short branches separating these lineages (Fig. 2, Supplementary Figs. 1–3), we tested the hypothesis of ILS in the early evolutionary radiation of Ulvophyceae.

### Ancient incomplete lineage sorting explains topological conflict

Phylogenetic incongruence can result from multiple processes and factors that often act together, such as hybridization, ILS, gene duplication/loss, and model misspecification^36–40^. It is widely known that model misspecification can have a substantial impact on phylogenetic estimation, especially for ancient lineages like the Chlorophyta^41–43^. It has been suggested that conflicting phylogenetic relationships and the ubiquitous long branches within the Ulvophyceae resulted from inadequate modeling^3^. To address these problems and to test the performance of different models, we used three strategies in the concatenation-based analyses (i.e., partition strategy, PMSF model, and GHOST model). Substitution rate variation across genes, sites and lineages were all taken into account^44,45^. LBA was alleviated in our analyses by increasing taxon sampling and applying heterogeneous models, and most of the inferred topologies using different strategies were congruent (Fig. 2, Supplementary Figs. 1–3), which conferred confidence in the phylogenetic results. However, the phylogenetic position of the DS clade remained uncertain. Specifically, phylogenetic analysis based on a gene-wise partition and site-heterogeneous model supports congruent topology in which the TC clade diverged before the UUOI and DS clade (Supplementary Figs. S1, S2). In contrast, in the phylogenetic tree estimated by the heterotachy model, the UUOI clade was resolved as a sister to the two other clades (Supplementary Fig. 3). The different topologies imply that these models were insufficient to explain the phylogenetic conflict, and/or that our dataset was sensitive to the rate variation across lineages at deep divergences in the Ulvophyceae. It is difficult to directly measure the fit between these complex models and datasets in phylogenetic analyses. Some approaches for assessing model-to-data fitness have been commonly used for model validation, that is, simulation data are generated using the model and parameter values, and observed data are compared with the simulated data to determine if they can represent the distribution defined from the model^46,47^. Puttick et al.^48^ used posterior predictive tests and showed that the site-heterogeneous CATGTR model fitted better than the site-homogeneous GTR model for inferring land plant phylogeny. So far, however, methods of simulating the PMSF or General Heterogeneous evolution On a Single Topology (GHOST) model are not available, and future work is needed for evaluating model fitness.

ILS has been intensively studied in recently diverging lineages^49^, but there is no reason to rule out ILS in ancient rapid radiations, confounding the phylogenetic history of deep nodes, as has been demonstrated in early land plants relationships^50^, early angiosperm evolution^51,52^, and rapid adaptive radiations, including those of neoavian birds^53^, legumes^54^, and the amaranth family^37^. Under these circumstances, the multispecies coalescent method is expected to perform better than the concatenation method, since the former is able to deal with the gene tree heterogeneity caused by ILS, and is likely to result in a more accurate estimation of the species tree^31,32^. In our study, although the branching order of the UUOI, DS and TC clades was not well resolved in concatenation-based analyses, we recovered the same phylogeny in the concatenation-based analyses using a partition strategy and the PMSF model, as in the coalescent-based analysis (Fig. 2B). Moreover, several deep branches were very short, especially at the internodes of the UUOI-DS clade, and Bryopsidales–Chlorophyceae clade (Fig. 2A). Our analyses revealed strong gene tree conflicts among the focal branches, corresponding to the gene tree quartet probability observed in Fig. 3A. The proportions of genes supporting alternative topologies were roughly equal, similar to the observations reported by previous transcriptomic analyses^18^. These phenomena hint at the possibility of ILS, in conjunction with an ancient rapid radiation^42,51,55–57^. Our simulation results confirmed that deep divergences in the Ulvophyceae were affected by pervasive ILS. Consistent with several previous studies that found that the multispecies coalescent model is superior to concatenation in diverse phylogenomic datasets across the tree of life^40,51^, the multispecies coalescent model is likely to be suitable for our dataset in the presence of ILS, emphasizing that the degree of fit between model and datasets should be considered in phylogenetic analyses.

### Insights in ulvophycean evolution at the Mesoproterozoic–Neoproterozoic transition

Fossil calibration is by far the most critical factor in divergence time estimation^58^. Although the Phanerozoic fossils used in this study provide reasonably robust calibration points, they are relatively few in number owing to the scarce fossil records of green algae. This means that the Precambrian fossils used as calibration points potentially exert a strong influence on time estimation. In this context, it is critical to carefully assess the phylogenetic interpretations of the selected Precambrian fossils^59,60^, particularly given their relatively simple morphologies^61^. Del Cortona et al.^18^ have evaluated the impact of different phylogenetic assignments of the middle Neoproterozoic filamentous fossil *Proterocladus major* (ca. 750 Mya, ref. ^24^). Their study supported an early diversification of the Ulvophyceae in the Tonian Period, with further taxonomic, morphological, and ecological expansions in the Ediacaran and Ordovician Periods, consistent with the Paleozoic macroalgal fossil record^61,62^. The recent discovery of the one-billion-year-old fossil *Proterocladus antiquus* provided an opportunity to further evaluate how these ancient fossils impact our understanding of the timescale of green algal evolution^28^. Based on morphological similarities with contemporary species of Cladophorales, *P. antiquus* has been interpreted as a total-group cladophoralean^28^. This interpretation is credible given the apparent coenocytic organization of *Proterocladus*, the fact that most of modern coenocytic green algae belong to the Cladophorales, and other morphological features of *Proterocladus* (e.g., a rather unusual branching style, holdfast) that are consistent with a cladophoralean interpretation. However, it is impossible to exclude the possibility of convergent evolution. Coenocytic forms are not restricted to the Cladophorales, and also occur in the Ulotrichales (e.g., *Acrosiphonia*), for example. It is thus reasonable to assume that during the long evolutionary history of the green algae there have been other—perhaps extinct—lineages of Ulvophyceae with similar coenocytic architectures. A more conservative strategy would thus be to treat *Proterocladus* as a total-group ulvophycean. Finally, there is a remote possibility that *Proterocladus* belongs to an extinct coenocytic lineage outside the Ulvophyceae or even outside the green algae, considering that coenocytic cells are present in diverse and unrelated groups of algae, including Xanthophyceae (e.g., *Vaucheria*), red algae (e.g., *Griffithsia*), even though their morphology differs significantly from *Proterocladus* or Cladophorales green algae. These different phylogenetic interpretations of *P. antiquus* led us to evaluate the impact of three different taxonomic assignments of *P. antiquus* on divergence time estimates. Note that Sforna et al.^63^ recently reported a ~1 Gyr-old multicellular photosynthetic eukaryote, *Arctacellularia tetragonala*, with a siphonocladous organization, but that the alga was not associated to either green or red algae.

The time-calibrated trees inferred using the first (assignment of *P. antiquus* as a stem-group Cladophorales) and second (assignment of *P. antiquus* as a stem-group Ulvophyceae s.s.) calibration strategies indicated an origin and early diversification of the core Chlorophyta in the Mesoproterozoic, and early diversification of the Ulvophyceae in the late Mesoproterozoic and early Neoproterozoic. Although these estimates are older than what has been reported in previous studies^18,64^, they are largely congruent with current understanding of eukaryotic divergence times^65–67^, and some previous hypotheses on green algal evolution^68^. In addition, besides *P. antiquus*, a number of other paleontological studies supported an earlier diversification of ulvophyte lineages, including the discovery of unbranched filamentous macrofossils of possibly cladophoralean affinity from the early Tonian Period^69^, and the tentative interpretation of the Tonian macrofossil *Tawuia* as a coenocytic green macroalga^70^. Together, these data indicate that green macroalgae may have already colonized marine benthic environments by the early Neoproterozoic, and possibly earlier in the Mesoproterozoic^61^, although they may have been ecologically restricted due to severe nutrient limitation^71^. The third calibration strategy (excluding *P. antiquus*) produced time estimates close to those in Del Cortona et al.^18^ supporting a Stenian-Tonian origin of Ulvophyceae s.s. and diversification of its lineages in the late Tonian/Cryogenian periods.

By regarding the different time estimates as plausible evolutionary scenarios, our time-calibrated phylogenies provide a framework for understanding green algal evolution and their impact on the past climate and paleogeology. Since ulvophyceans mainly consist of marine macroalgae, their diversification may have paved the road for significant changes in the global carbon cycle and oceanic redox structure at the Mesoproterozoic–Neoproterozoic transition and onwards^72–74^. In light of the current results, it is intriguing to note that, although there is evidence for oxygenated bottom waters in Mesoproterozoic oceans^75–78^, geochemical data indicate a fundamental restructuring of oceanic redox architectures in the Neoproterozoic^72,79^. Biomarker fossils also allude to an ecological rise of green algae in the Cryogenian Period^73^, necessitating the divergence of the chlorophytes prior to the Cryogenian. The evolutionary timeline of chlorophytes and ulvophyceans presented here is consistent with these geochemical and biomarker data, although a more nuanced correlation between molecular clocks and sedimentary rocks will require more refined phylogenetic, paleontological, and geochemical data to be acquired in the future.

In conclusion, this study furthers our understanding on phylogenetic relationships and the timescale of ulvophycean diversification based on a large-scale nuclear dataset. Our study is based on an extensive nuclear gene sampling (including 11 newly sequenced ulvophycean transcriptomes) and provides a robust phylogenetic framework for studying the evolution of ulvophyceans. In concordance with previous studies, our analysis did not support monophyly of the Ulvophyceae. The branching order within the Ulvophyceae s.s. was found to be sensitive to the rate variation across lineages. Gene tree discordance together with coalescent simulations and model fitting indicates that ILS was a possible explanation of topological conflicts. The recent discovery of the one-billion-year-old fossil *Proterocladus antiquus* offers an opportunity to test its impact on the estimation of ulvophycean divergence times. Taking into account different phylogenetic assignments of the new fossil, our molecular clock analyses indicate that the Ulvophyceae s.s. may have diverged in the late Mesoproterozoic to early Neoproterozoic. The early divergence and diversification of green seaweeds hint at the possibility of geobiological roles of chlorophytes and ulvophyceans in environmental changes in the Mesoproterozoic and Neoproterozoic Era.

## Methods

### Taxon sampling and culture conditions

Eleven cultured strains of Ulvophyceae were used in this study, including previously unsampled species from the Dasycladales and Cladophorales, and lineages with controversial phylogenetic relationships (e.g., Bryopsidales and Scotinosphaerales). *Bryopsis hypnoides* (SAG 7.86), *Ostreobium quekettii* (SAG 6.99), *Rhexinema paucicellulare* (SAG 463-1), *Hazenia basiliensis* (SAG 466-2) and *Scotinosphaera austriaca* (SAG 48.96) strains were obtained from the Culture Collection of Algae at the University of Göttingen, Germany (SAG). *Acetabularia calyculus* (UTEX LB 2702), *A. kilneri* (UTEX LB 2696), *Polyphysa clavata* (UTEX LB 2708) and *Chlorocladus australasicus* (UTEX LB 2686) cultures were accessed from the Culture Collection of Algae at the University of Texas at Austin, USA (UTEX). *Chlorocladiella pisiformis* (HBI-BN1708) and *Chlorocladiella medogensis* (HBI-TB1640) were obtained from the Freshwater Algal Herbarium, Institute of Hydrobiology, Chinese Academy of Science, Wuhan, China (HBI). These 11 strains have expanded the limited taxon sampling of the Ulvophyceae in previous studies. All strains were cultured at 20 °C, 12-h light/12-h dark cycle with a photon flux density of 30 μmol  photons m^−2^ s^−1^.

### Transcriptome sequencing, assembly, and dataset retrieval

According to the cultivation conditions and growth period of the strains, we cultivated the strains for 1 to 2 months during its logarithmic growth period. In order to obtain as many expressed genes as possible, the logarithmic phase strains were enriched at three time points (9:00, 15:00, 21:00) by centrifugation. The collected strains were immediately flash frozen in liquid nitrogen and stored in a −80 °C refrigerator until RNA extraction. Total RNA was extracted using TRIzol reagent (Ambion) according to the manufacturer’s instructions. The RNA libraries were generated through 150 bp paired-end reads (PE150) sequencing using the Illumina Novaseq platform, at Novogene Bioinformatics Technology Co., Ltd. (Beijing, China). Clean data were obtained by removing reads containing adapter or ploy-N, and low-quality reads from the raw data. The contigs were de novo assembled with Trinity (ref. ^80^) using a k-mer = 25, and clustered using Corset^81^ with default parameters. The longest contig in each cluster was chosen as a unigene for further analyses. The full dataset was derived from eight published genomes and 50 published transcriptomes, in addition to the 11 newly sequenced transcriptomes (Supplementary Data 1).

### Ortholog selection, alignment, and trimming

We obtained an initial dataset of 1564 single copy orthologs using OrthoMCL v2.0.9 (ref. ^82^) with default parameters, from seven published genomes of Ulvophyceae, Chlorophyceae and Trebouxiophyceae (*Ulva mutabilis*, *Caulerpa lentillifera*, *Chlamydomonas reinhardtii*, *Dunaliella salina*, *Volvox carteri*, *Chlorella variabilis* and *Coccomyxa subellipsoidea*) (Supplementary Data 1). The resulting 1564 clusters of orthologous groups (COGs) were used as references in Orthograph v0.6.3 (ref. ^83^) to select orthologous genes (OGs) from other 62 genomic/transcriptomic data.

Amino acid sequences of each OG were aligned by MAFFT v7.310 (ref. ^84^) using the L-INS-I algorithm. Ambiguously aligned regions were excluded using Gblocks v0.91b (ref. ^85^) with half gaps allowed. The short sequences were removed using trimAl v1.2 (ref. ^86^) with parameters: resoverlap 0.5 and seqoverlap 50. We further filtered the sequences with taxon occupancy below 80% and length <100 bp, resulting in a dataset of 884 OGs for downstream phylogenetic analysis.

### Phylogenetic inferences

The dataset of 884 OGs was analyzed using coalescent and concatenation approaches. For the coalescent approach, ML gene trees were built using IQ-TREE v1.6.12 (ref. ^87^) with automatic selection of the best-fit substitution model for each gene. Branch support was evaluated with 1,000 replicates of the ultrafast bootstrap^88^. To minimize potential impacts of gene tree estimation error for species tree reconstruction, we collapsed low support branches (bootstrap support <20%) from gene trees with Newick Utilities v1.6 (ref. ^89^). The species tree was inferred using ASTRAL v5.7.3 (ref. ^90^) with support values estimated by local posterior probabilities and multi-locus bootstrapping (gene and site resampling). The concatenation analyses were performed using IQ-TREE v1.6.12 with three settings: (1) the gene-wise partitioned analysis using the best-fit model estimated to each partition, (2) the phylogenetic analysis performing the PMSF model with LG + C20 + F + Γ4 and a guide tree specified^45^, and (3) the heterotachy analysis using the GHOST model^44^. Branch support in each analysis was estimated with 1000 ultrafast bootstrap and SH-aLRT branch test replicates^91^.

### Ancestral state reconstruction

In order to reconstruct the ancestral state of cytomorphological traits of the Ulvophyceae, we recoded the cell types of 69 species: unicellular (0), colonial (1), siphonous (2), multicellular (3), siphonocladous (4), and all cytomorphological traits were obtained from the literature and AlgaeBase^92^. The coalescent-based phylogeny was used to guide the ancestral state reconstruction. The ancestral state of the cell type was reconstructed using the Mk1 model (Markov k-state 1 parameter model), implemented under a maximum-likelihood framework in the Mesquite v3.61 (http://www.mesquiteproject.org).

### Simulation of incomplete lineage sorting (ILS)

Gene tree concordance and conflicts were quantified using PhyParts (ref. ^36^), mapping against the coalescent species tree, with 50% bootstrap support threshold of individual gene trees (-s parameter). The pie charts of each node were summarized and generated by PhyPartsPieCharts and ETE3 (https://github.com/mossmatters/MJPythonNotebooks, ref. ^93^). Normalized quartet supports of the internal branches in the species tree were performed in ASTRAL v5.7.3 with the parameter “-t 8”.

To explore whether gene tree conflicts around two short internal branches can be explained by ILS, we carried out ILS simulations and correlation test following the approach of refs. ^37,52,94^. The gene trees are simulated under the multispecies coalescent model from the ASTRAL species tree, and then the correlation between the observed frequencies and the simulated frequencies of gene trees is calculated. If there is considerable agreement between simulated and observed gene trees (the correlation coefficient is close to: (1), ILS is likely to occur and may well account for gene tree conflicts. First, our OGs dataset of 69 taxa was filtered to two small datasets of 7 taxa, referred herein as 7-taxon(I) and 7-taxon(II). The 7-taxon(I) dataset was selected around the internal branch between the UUOI (i.e., Ulotrichales, Ulvales, Oltmannsiellopsidales, and Ignatiales) and DS (i.e., Dasycladales and Scotinosphaerales) clades (Fig. 2A), and the 7-taxon(II) dataset was selected around Bryopsidales and Chlorophyceae. Amino acid sequences of OGs of each 7-taxon dataset were aligned using MAFFT v7.310 (ref. ^84^) with the L-INS-I algorithm, and alignments were trimmed using Gblocks v0.91b (ref. ^85^) with ‘Allowed Gap Positions set to half’. OGs with taxon occupancy rate of <80% and length of <100 bp were removed, resulting in 891 genes for the 7-taxon(I) and 1280 genes for the 7-taxon(II) dataset. ML gene trees for each 7-taxon dataset were reconstructed using IQ-TREE v1.6.12 with the best-fit model estimated for each gene. Both species trees were estimated using ASTRAL v5.7.3, with internal branch lengths assigned in coalescent units and terminal branch lengths aligned. We simulated 100,000 gene trees for both datasets under the multispecies coalescent model using the function sim.coaltree.sp in the R package Phybase v1.5 (ref. ^95^). To explore the correlation between the gene trees as estimated by the multispecies coalescent model and the observed gene trees, we calculated the topological frequencies of the observed gene trees and the simulated gene trees for each 7-taxon dataset. The correlation between the observed frequencies and the simulated frequencies of gene trees was performed using the cor.test function in R.

### Divergence time estimation

#### Dataset assembly for molecular dating

Divergence times were estimated for the dataset of 884 OGs using the program MCMCTree in the PAML package v4.9j (ref. ^96^). We identified the clock-likeliness of each gene using SortaDate^97^ by the three criteria sequentially: 3 = least topological conflict against the coalescent species tree, 1 = minimal root-to-tip variance and 2 = tree length (discernible information content). The 200 most clock-like genes were selected for the molecular clock analyses. The approximate likelihood calculations in MCMCTree were implemented using CODEML under the LG + Γ_4_ + F model^98^. The MCMC process was run for 10 million generations sampled every 1000 generations after a burnin of 1 million iterations. We ran two independent chains to check for convergence and confirmed that the effective sample sizes (ESS) of all parameters were above 200 using Tracer v1.7.1 (ref. ^99^).

#### Rate priors

We used a time unit of 100 My (million years) and an independent rate (IR) model in our divergence time estimation. For rate priors, we calculated the amino acid pairwise distance between *Ulva mutabilis* and *Chlorocladiella pisiformis* (0.3581 substitutions per site) using the LG + Γ_4_ + F model in CODEML (ref. ^96^). The divergence time between the two species was represented with the fossil-based age ~1000 Ma (ref. ^28^), which indicated that the mean rate for branches (*μ*) was assigned a gamma hyperprior G (2, 55.85) with mean 2/55.85 = 0.03581 substitutions per site per time unit (100 My). The variance of branch rates (*σ*^2^) was assigned G (1, 10) with mean 0.1.

#### Time priors

The time prior for all nodes was generated by combining fossil calibrations and the birth-death process. Because of the relatively small number of fossil occurrences that can be reliably used as calibration points, we needed more informative parameters for the birth-death model in our calibration-poor phylogeny. The birth rate *λ* and death rate *μ* were estimated by using the data-driven birth-death (ddBD) tree prior in R^100^. The sampling fraction *ρ* was the proportion of our ingroup sample size (65 taxa) to the number of extant species in Ulvophyceae, Chlorophyceae and Trebouxiophyceae (~6478) (https://www.algaebase.org). To ensure our priors on divergence times were appropriate, we ran the MCMC analyses without molecular data to obtain the effective priors.

#### Fossil constraints

We applied node age constraints to eight nodes using fossil information and secondary calibrations (Table 1). Node 1 was set with a uniform distribution with a hard minimum age (*p*_*L*_ = 1e−300) and a soft maximum age (*p*_*U*_ = 0.025). Nodes 5 and 8 were set with uniform distributions with a soft minimum age (*p*_*L*_ = 0.025) and a soft maximum age (*p*_*U*_ = 0.025) as they were secondary calibrations. The other nodes were set as truncated Cauchy distributions with a hard minimum bound (*p*_*L*_ = 1e−300). Three strategies were applied to test the impact of the one-billion-year-old fossil *Proterocladus antiquus* in our dating analyses. From strategy 1–3, we respectively assigned *Proterocladus antiquus* to the stem Cladophorales (node 6 − 1), stem Ulvophyceae s.s. (node 6 − 2), and excluded this fossil (node 6 − 3). Calibration densities of the effective prior and posterior distributions were plotted by MCMCTreeR v1.1 in ref. ^101^.

**Table 1:** Detailed information of the calibration nodes.

Node	Clade	Fossils	Node calibration	Time prior (My)	Reference
1	UTC^a^ clade + Chlorodendrophyceae	NA	root	1891-1000	Refs. ^28,102^
2	Trebouxiophyceae	*Botryococcus* sp.	*Botryococcus* stem	Min 298.75	Ref. ^103^
3	Chlorophyceae	*Scenedesmus bifidus*	*Scenedesmus* stem	Min 125	Ref. ^104^
4	Ulvophyceae	*Buthograptus laxus*	Bryopsidales stem	Min 452.3	Ref. ^25^
5	Ulvophyceae	secondary calibration	Bryopsidales crown	533-425	Ref. ^26^
6-1	Ulvophyceae	*Proterocladus antiquus*	Cladophorales stem	Min 1000	Ref. ^28^
6-2	Ulvophyceae	*Proterocladus antiquus*	Ulvophyceae s.s stem	Min 1000	Ref. ^28^
6-3	NA	NA	NA	NA	NA
7	Ulvophyceae	*Chaetocladus dubius*	Dasycladales stem	Min 443.8	Ref. ^25^
8	Ulvophyceae	secondary calibration	Dasycladales crown	517-407	Ref. ^26^

Node numbers refer to the calibration points in Supplementary Figs. 6, 7, 8.

^a^*UTC* Ulvophyceae, Chlorophyceae, and Trebouxiophyceae.

### Reporting summary

Further information on research design is available in the Nature Research Reporting Summary linked to this article.

## Supplementary information

Supplementary Information

Description of Additional Supplementary Files

Supplementary Data 1

Reporting Summary

## Data Availability

The raw Illumina data generated for this study are available through the Sequence Read Archive (SRA accession PRJNA726747). The alignment data and phylogenetic trees are available from Figshare: https://figshare.com/s/40126faad551cdfccf69. Source data are provided with this paper.

## Source data

Source Data

## References

[1] Brodie, J., Maggs, C. A., & John, D. M. Green seaweeds of Britain and Ireland. pp. 242 (British Phycological Society, 2007).

[2] Leliaert F (2012). Phylogeny and molecular evolution of the green algae. Crit. Rev. Plant Sci..

[3] Del Cortona A, Leliaert F (2018). Molecular evolution and morphological diversification of ulvophytes (Chlorophyta). Perspect. Phycol..

[4] Fang L, Leliaert F, Zhang Z, Penny D, Zhong B (2017). Evolution of the Chlorophyta: Insights from chloroplast phylogenomic analyses. J. Syst. Evol..

[5] Prazukin AV, Anufriieva EV, Shadrin NV (2020). Is biomass of filamentous green algae *Cladophora* spp. (Chlorophyta, Ulvophyceae) an unlimited cheap and valuable resource for medicine and pharmacology? A review. Rev. Aquacult.

[6] Cocquyt E, Verbruggen H, Leliaert F, De Clerck O (2010). Evolution and cytological diversification of the green seaweeds (Ulvophyceae). Mol. Biol. Evol..

[7] Atkinson N (2016). Introducing an algal carbon-concentrating mechanism into higher plants: location and incorporation of key components. Plant Biotechnol. J..

[8] Mattox, K. R. & Stewart, K. D. Classification of the green algae: a concept based on comparative cytology. In: Irvine, D. E. G. & John, D. M., editors. Systematics of the Green Algae. pp. 29–72 (Academic Press, 1984).

[9] O’Kelly, C. J. & Floyd, G. L. Correlations among patterns of sporangial structure and development, life histories, and ultrastructural features in the Ulvophyceae. In: *Systematics of the Green Algae* (eds Irvine, D. E. G. & John, D. M.) pp. 121–156 (Academic Press, 1984).

[10] Van den Hoek C, Stam WT, Olsen JL (1988). The emergence of a new chlorophytan system, and Dr. Kornmann’s contribution thereto. Helgol. Mar. Res..

[11] Watanabe S, Nakayama T (2007). Ultrastructure and phylogenetic relationships of the unicellular green algae *Ignatius tetrasporus* and *Pseudocharacium americanum* (Chlorophyta). Phycol. Res..

[12] Škaloud P, Kalina T, Nemjova K, De Clerck O, Leliaert F (2013). Morphology and phylogenetic position of the freshwater green microalgae *Chlorochytrium* (Chlorophyceae) and *Scotinosphaera* (Scotinosphaerales, ord. nov., Ulvophyceae). J. Phycol..

[13] Fučíková K (2014). New phylogenetic hypotheses for the core Chlorophyta based on chloroplast sequence data. Front. Ecol. Evol..

[14] Leliaert F, López-Bautista JM (2015). The chloroplast genomes of *Bryopsis plumosa* and *Tydemania expeditiones* (Bryopsidales, Chlorophyta): compact genomes and genes of bacterial origin. BMC Genom..

[15] Fang L (2018). Improving phylogenetic inference of core Chlorophyta using chloroplast sequences with strong phylogenetic signals and heterogeneous models. Mol. Phylogenet. Evol..

[16] Sun L (2016). Chloroplast phylogenomic inference of green algae relationships. Sci. Rep..

[17] Li X (2021). Large phylogenomic datasets reveal deep relationships and trait evolution in chlorophyte green algae. Genome Biol. Evol.

[18] Del Cortona A (2020). Neoproterozoic origin and multiple transitions to macroscopic growth in green seaweeds. Proc. Natl Acad. Sci. U.S.A..

[19] Leebens-Mack JH (2019). One thousand plant transcriptomes and the phylogenomics of green plants. Nature.

[20] Gulbrandsen ØS, Andresen IJ, Krabberød AK, Bråte J, Shalchian-Tabrizi K (2021). Phylogenomic analysis restructures the Ulvophyceae. J. Phycol..

[21] Melton JR, Leliaert F, Tronholm A, Lopez-Bautista JM (2015). The complete chloroplast and mitochondrial genomes of the green macroalga *Ulva* sp. UNA00071828 (Ulvophyceae, Chlorophyta). PLoS ONE.

[22] Javaux EJ, Knoll AH (2017). Micropaleontology of the lower Mesoproterozoic Roper Group, Australia, and implications for early eukaryotic evolution. J. Paleontol..

[23] Moczydłowska M, Landing ED, Zang W, Palacios T (2011). Proterozoic phytoplankton and timing of chlorophyte algae origins. Palaeontology.

[24] Butterfield NJ, Knoll AH, Swett K (1994). Paleobiology of the Neoproterozoic Svanbergfjellet Formation, Spitsbergen. Lethaia.

[25] LoDuca ST (2018). New Ordovician marine macroalgae from North America, with observations on *Buthograptus*, *Callithamnopsis*, and *Chaetocladus*. J. Paleontol..

[26] Verbruggen H (2009). A multi-locus time-calibrated phylogeny of the siphonous green algae. Mol. Phylogenet. Evol..

[27] Butterfield NJ (2009). Modes of pre-Ediacaran multicellularity. Precambrian Res..

[28] Tang Q, Pang K, Yuan X, Xiao S (2020). A one-billion-year-old multicellular chlorophyte. Nat. Ecol. Evol..

[29] Graham LE (2019). Digging deeper: why we need more Proterozoic algal fossils and how to get them. J. Phycol..

[30] Xiao S, Tang Q (2018). After the boring billion and before the freezing millions: evolutionary patterns and innovations in the Tonian Period. Emerg. Top. Life Sci..

[31] Rannala, B., Edwards, S. V., Leaché, A. & Yang, Z. The multi-species coalescent model and species tree inference. *Phylogenet. Genom. Era* book section 3.3, pp. 3.3:1–21 (2020).

[32] Liu L, Wu S, Yu L (2015). Coalescent methods for estimating species trees from phylogenomic data. J. Syst. Evol..

[33] Turmel M, Otis C, Lemieux C (2017). Divergent copies of the large inverted repeat in the chloroplast genomes of ulvophycean green algae. Sci. Rep..

[34] Leliaert F (2009). Systematics of the marine microfilamentous green algae *Uronema curvatum* and *Urospora microscopica* (Chlorophyta). Eur. J. Phycol..

[35] Del Cortona A (2017). The plastid genome in Cladophorales green algae is encoded by hairpin chromosomes. Curr. Biol..

[36] Smith SA, Moore MJ, Brown JW, Yang Y (2015). Analysis of phylogenomic datasets reveals conflict, concordance, and gene duplications with examples from animals and plants. BMC Evol. Biol..

[37] Morales-Briones DF (2021). Disentangling sources of gene tree discordance in phylogenomic data sets: testing ancient hybridizations in Amaranthaceae s.l. Syst. Biol..

[38] Mirarab S, Bayzid MS, Warnow T (2016). Evaluating summary methods for multilocus species tree estimation in the presence of incomplete lineage sorting. Syst. Biol..

[39] Pease JB, Brown JW, Walker JF, Hinchliff CE, Smith SA (2018). Quartet sampling distinguishes lack of support from conflicting support in the green plant tree of life. Am. J. Bot..

[40] Jiang X, Edwards SV, Liu L (2020). The multispecies coalescent model outperforms concatenation across diverse phylogenomic data sets. Syst. Biol..

[41] Blom M, Bragg JG, Potter S, Moritz C (2017). Accounting for uncertainty in gene tree estimation: summary-coalescent species tree inference in a challenging radiation of Australian lizards. Syst. Biol..

[42] Liu L, Xi Z, Davis CC (2015). Coalescent methods are robust to the simultaneous effects of long branches and incomplete lineage sorting. Mol. Biol. Evol..

[43] Philippe H (2017). Pitfalls in supermatrix phylogenomics. Eur. J. Taxon..

[44] Crotty SM (2020). GHOST: recovering historical signal from heterotachously evolved sequence alignments. Syst. Biol..

[45] Wang HC, Minh BQ, Susko E, Roger AJ (2018). Modeling site heterogeneity with posterior mean site frequency profiles accelerates accurate phylogenomic estimation. Syst. Biol..

[46] Brown JM, Thomson RC (2018). Evaluating model performance in evolutionary biology. Annu. Rev. Ecol. Evol. Syst..

[47] Foster PG (2004). Modeling compositional heterogeneity. Syst. Biol..

[48] Puttick MN (2018). The interrelationships of land plants and the nature of the ancestral embryophyte. Curr. Biol..

[49] Edwards SV (2009). Is a new and general theory of molecular systematics emerging?. Evolution.

[50] Zhong B, Liu L, Yan Z, Penny D (2013). Origin of land plants using the multispecies coalescent model. Trends Plant Sci..

[51] Yang L (2020). Phylogenomic insights into deep phylogeny of angiosperms based on broad nuclear gene sampling. Plant Commun..

[52] Yang Y (2020). Prickly waterlily and rigid hornwort genomes shed light on early angiosperm evolution. Nat. Plants.

[53] Suh A, Smeds L, Ellegren H (2015). The dynamics of incomplete lineage sorting across the ancient adaptive radiation of Neoavian birds. PLoS Biol..

[54] Koenen E (2020). Large-scale genomic sequence data resolve the deepest divergences in the legume phylogeny and support a near-simultaneous evolutionary origin of all six subfamilies. N. Phytol..

[55] Degnan JH, Rosenberg NA (2009). Gene tree discordance, phylogenetic inference and the multispecies coalescent. Trends Ecol. Evol..

[56] Song S, Liu L, Edwards SV, Wu S (2012). Resolving conflict in eutherian mammal phylogeny using phylogenomics and the multispecies coalescent model. Proc. Natl Acad. Sci. U.S.A..

[57] Cloutier A (2019). Whole-genome analyses resolve the phylogeny of flightless birds (Palaeognathae) in the presence of an empirical anomaly zone. Syst. Biol..

[58] Sauquet H (2013). A practical guide to molecular dating. C. R. Palevol..

[59] Ho SYW, Phillips MJ (2009). Accounting for calibration uncertainty in phylogenetic estimation of evolutionary divergence times. Syst. Biol..

[60] Parham JF (2012). Best practices for justifying fossil calibrations. Syst. Biol..

[61] Bykova N (2020). Seaweeds through time: morphological and ecological analysis of Proterozoic and early Paleozoic benthic macroalgae. Precambrian Res.

[62] LoDuca ST, Bykova N, Wu M, Xiao S, Zhao Y (2017). Seaweed morphology and ecology during the great animal diversification events of the early Paleozoic: a tale of two floras. Geobiology.

[63] Sforna MC (2022). Intracellular bound chlorophyll residues identify 1 Gyr-old fossils as eukaryotic algae. Nat. Commun..

[64] Jackson C, Knoll AH, Chan CX, Verbruggen H (2018). Plastid phylogenomics with broad taxon sampling further elucidates the distinct evolutionary origins and timing of secondary green plastids. Sci. Rep..

[65] Strassert JFH, Irisarri I, Williams TA, Burki F (2021). A molecular timescale for eukaryote evolution with implications for the origin of red algal-derived plastids. Nat. Commun..

[66] Nie Y (2020). Accounting for uncertainty in the evolutionary timescale of green plants through clock-partitioning and fossil calibration strategies. Syst. Biol..

[67] Sánchez-Baracaldo P, Raven JA, Pisani D, Knoll AH (2017). Early photosynthetic eukaryotes inhabited low-salinity habitats. Proc. Natl Acad. Sci. U.S.A.

[68] Teyssèdre, B. Are the green algae (phylum Viridiplantae) two billion years old? *Carnets Géol.* ***3***, CG2006 _A03 (2006).

[69] Maloney K (2021). New multicellular marine macroalgae from the early Tonian of northwestern Canada. Geology.

[70] Tang Q (2021). The Proterozoic macrofossil *Tawuia* as a coenocytic eukaryote and a possible macroalga. Palaeogeogr. Palaeoclimatol. Palaeoecol..

[71] Ozaki K, Reinhard CT, Tajika E (2019). A sluggish mid-Proterozoic biosphere and its effect on Earth’s redox balance. Geobiology.

[72] Guilbaud R, Poulton SW, Butterfield NJ, Zhu M, Shields-Zhou GA (2015). A global transition to ferruginous conditions in the early Neoproterozoic oceans. Nat. Geosci..

[73] Brocks J (2017). The rise of algae in Cryogenian oceans and the emergence of animals. Nature.

[74] Lyons TW, Reinhard CT, Planavsky NJ (2014). The rise of oxygen in Earth’s early ocean and atmosphere. Nature.

[75] Wang X (2017). Oxygen, climate and the chemical evolution of a 1400 million year old tropical marine setting. Am. J. Sci..

[76] Zhang K (2018). Oxygenation of the Mesoproterozoic ocean and the evolution of complex eukaryotes. Nat. Geosci..

[77] Sperling EA (2014). Redox heterogeneity of subsurface waters in the Mesoproterozoic ocean. Geobiology.

[78] Zhang S (2016). Sufficient oxygen for animal respiration 1,400 million years ago. Proc. Natl Acad. Sci. U.S.A..

[79] Planavsky NJ (2014). Low Mid-Proterozoic atmospheric oxygen levels and the delayed rise of animals. Science.

[80] Grabherr MG (2011). Full-length transcriptome assembly from RNA-Seq data without a reference genome. Nat. Biotechnol..

[81] Davidson NM, Oshlack A (2014). Corset: enabling differential gene expression analysis for *de novo* assembled transcriptomes. Genome Biol..

[82] Li L, Stoeckert CJ, Roos DS (2003). OrthoMCL: identification of ortholog groups for eukaryotic genomes. Genome Res.

[83] Petersen M (2017). Orthograph: a versatile tool for mapping coding nucleotide sequences to clusters of orthologous genes. BMC Bioinform..

[84] Katoh K, Standley DM (2013). MAFFT multiple sequence alignment software version 7: improvements in performance and usability. Mol. Biol. Evol..

[85] Castresana J (2000). Selection of conserved blocks from multiple alignments for their use in phylogenetic analysis. Mol. Biol. Evol..

[86] Capella-Gutiérrez S, Silla-Martinez JM, Gabaldon T (2009). trimAl: a tool for automated alignment trimming in large-scale phylogenetic analyses. Bioinformatics.

[87] Nguyen LT, Schmidt HA, von Haeseler A, Minh BQ (2015). IQ-TREE: a fast and effective stochastic algorithm for estimating maximum-likelihood phylogenies. Mol. Biol. Evol..

[88] Minh BQ, Nguyen MAT, von Haeseler A (2013). Ultrafast approximation for phylogenetic bootstrap. Mol. Biol. Evol..

[89] Junier T, Zdobnov EM (2010). The Newick utilities: high-throughput phylogenetic tree processing in the UNIX shell. Bioinformatics.

[90] Zhang C, Rabiee M, Sayyari E, Mirarab S (2018). ASTRAL-III: polynomial time species tree reconstruction from partially resolved gene trees. BMC Bioinform..

[91] Guindon S (2010). New algorithms and methods to estimate maximum-likelihood phylogenies: assessing the performance of PhyML 3.0. Syst. Biol..

[92] Guiry, M. D. & Guiry, G. M. AlgaeBase. World-wide electronic publication, National University of Ireland, Galway. https://www.algaebase.org (Accessed 22 March 2021).

[93] Huerta-Cepas J, Serra F, Bork P (2016). ETE 3: reconstruction, analysis, and visualization of phylogenomic data. Mol. Biol. Evol..

[94] Wang K (2018). Incomplete lineage sorting rather than hybridization explains the inconsistent phylogeny of the wisent. Commun. Biol..

[95] Liu L, Yu L (2010). Phybase: an R package for species tree analysis. Bioinformatics.

[96] Yang Z (2007). PAML 4: phylogenetic analysis by maximum likelihood. Mol. Biol. Evol..

[97] Smith SA, Brown JW, Walker JF (2018). So many genes, so little time: a practical approach to divergence-time estimation in the genomic era. PLoS ONE.

[98] Reis M, Yang Z (2011). Approximate likelihood calculation on a phylogeny for Bayesian estimation of divergence times. Mol. Biol. Evol..

[99] Rambaut A, Drummond AJ, Xie D, Baele G, Suchard MA (2018). Posterior summarization in Bayesian phylogenetics using Tracer 1.7. Syst. Biol..

[100] Tao Q, Barba-Montoya J, Kumar S (2021). Data-driven speciation tree prior for better species divergence times in calibration-poor molecular phylogenies. Bioinformatics.

[101] Puttick MN (2019). MCMCtreeR: functions to prepare MCMCtree analyses and visualize posterior ages on trees. Bioinformatics.

[102] Lamb DM, Awramik SM, Chapman DJ, Zhu S (2009). Evidence for eukaryotic diversification in the ∼1800 million-year-old Changzhougou Formation, North China. Precambrian Res..

[103] Colbath GK, Grenfell HR (1995). Review of biological affinities of Paleozoic acid-resistant, organic-walled eukaryotic algal microfossils (including “acritarchs”). Rev. Palaeobot. Palynol..

[104] Nye E, Feist-Burkhardt S, Horne DJ, Ross AJ, Whittaker JE (2008). The palaeoenvironment associated with a partial Iguanodon skeleton from the Upper Weald Clay (Barremian, Early Cretaceous) at Smokejacks Brickworks (Ockley, Surrey, UK), based on palynomorphs and ostracods. Cretac. Res.

[105] Škaloud, P., Rindi, F., Boedeker, C. & Leliaert, F. Freshwater Flora of Central Europe, Vol 13: Chlorophyta: Ulvophyceae. pp. 288 (Springer Spektrum, 2018).

[106] Darienko T, Rad-Menéndez C, Campbell CN, Pröschold T (2021). Molecular phylogeny of unicellular marine coccoid green algae revealed new insights into the systematics of the Ulvophyceae (Chlorophyta). Microorganisms.

