# Supplementary material for: Phylotranscriptomic insights into a Mesoproterozoic–Neoproterozoic origin and early radiation of green seaweeds (Ulvophyceae)
Source: Nat Commun. 2022 Mar 22;13:1610. doi: 10.1038/s41467-022-29282-9 (PMC8941102; doi:10.1038/s41467-022-29282-9)
Supplement: Supplementary file 1 — Supplementary Information [file 41467_2022_29282_MOESM1_ESM.pdf]

Supplementary Information

Phylotranscriptomic insights into a Mesoproterozoic-Neoproterozoic origin and early radiation of green seaweeds (Ulvophyceae)

Hou et al.

Supplementary Figures 1-10

Supplementary Note 1

Supplementary Table 1

Supplementary References

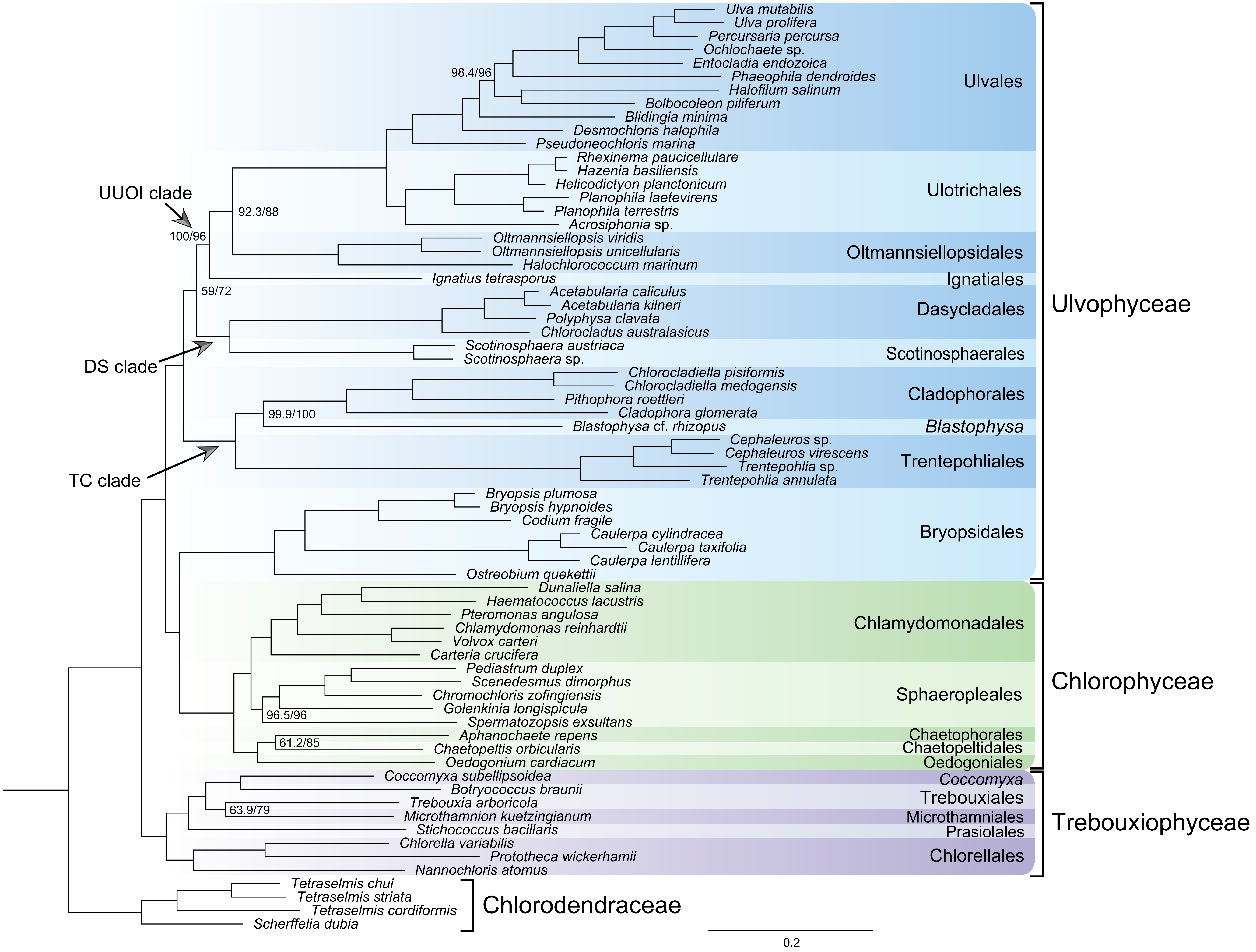

Supplementary Figure 1 Phylogenetic relationship of Ulvophyceae based on the concatenation approach and gene-wise partition. Support for each node is provided by SH-aLRT test and ultrafast bootstrap. Nodes without values indicate full supports.

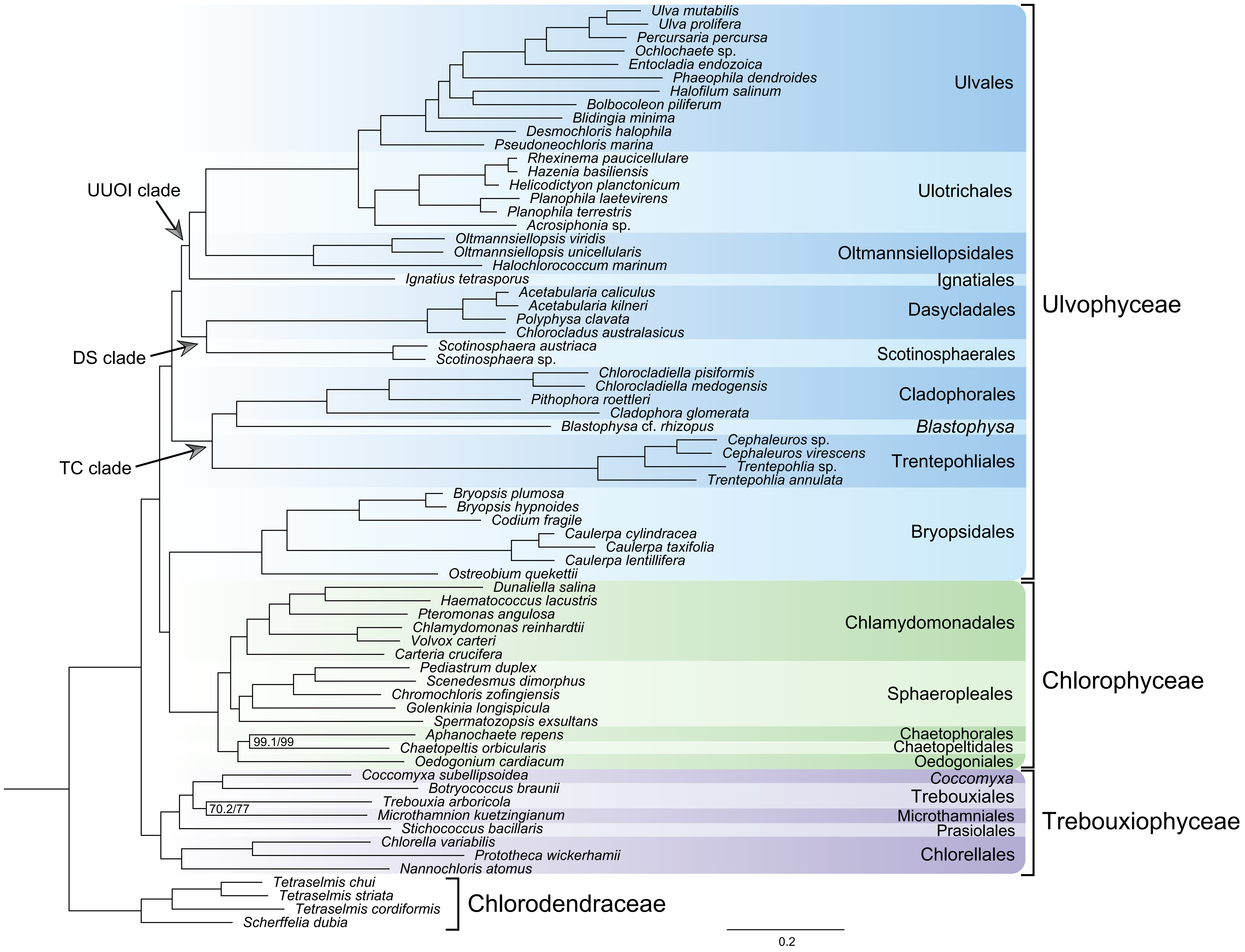

Supplementary Figure 2 Phylogenetic relationship of Ulvophyceae based on the concatenation approach and PMSF model. Support for each node is provided by SH-aLRT test and ultrafast bootstrap. Nodes without values indicate full supports.

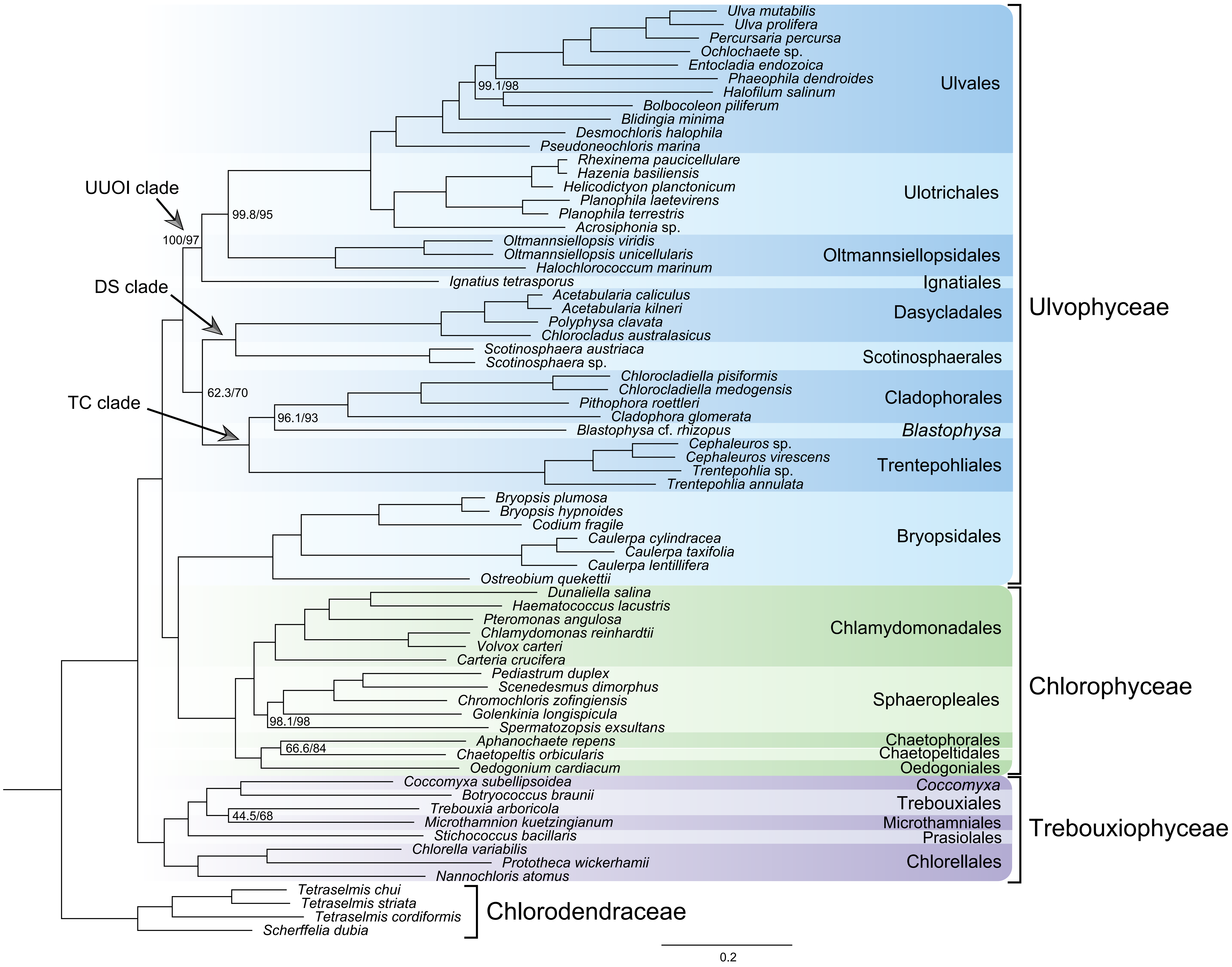

Supplementary Figure 3 Phylogenetic relationship of Ulvophyceae based on the concatenation approach and GHOST model. Support for each node is provided by SH-aLRT test and ultrafast bootstrap. Nodes without the value indicate full supports.

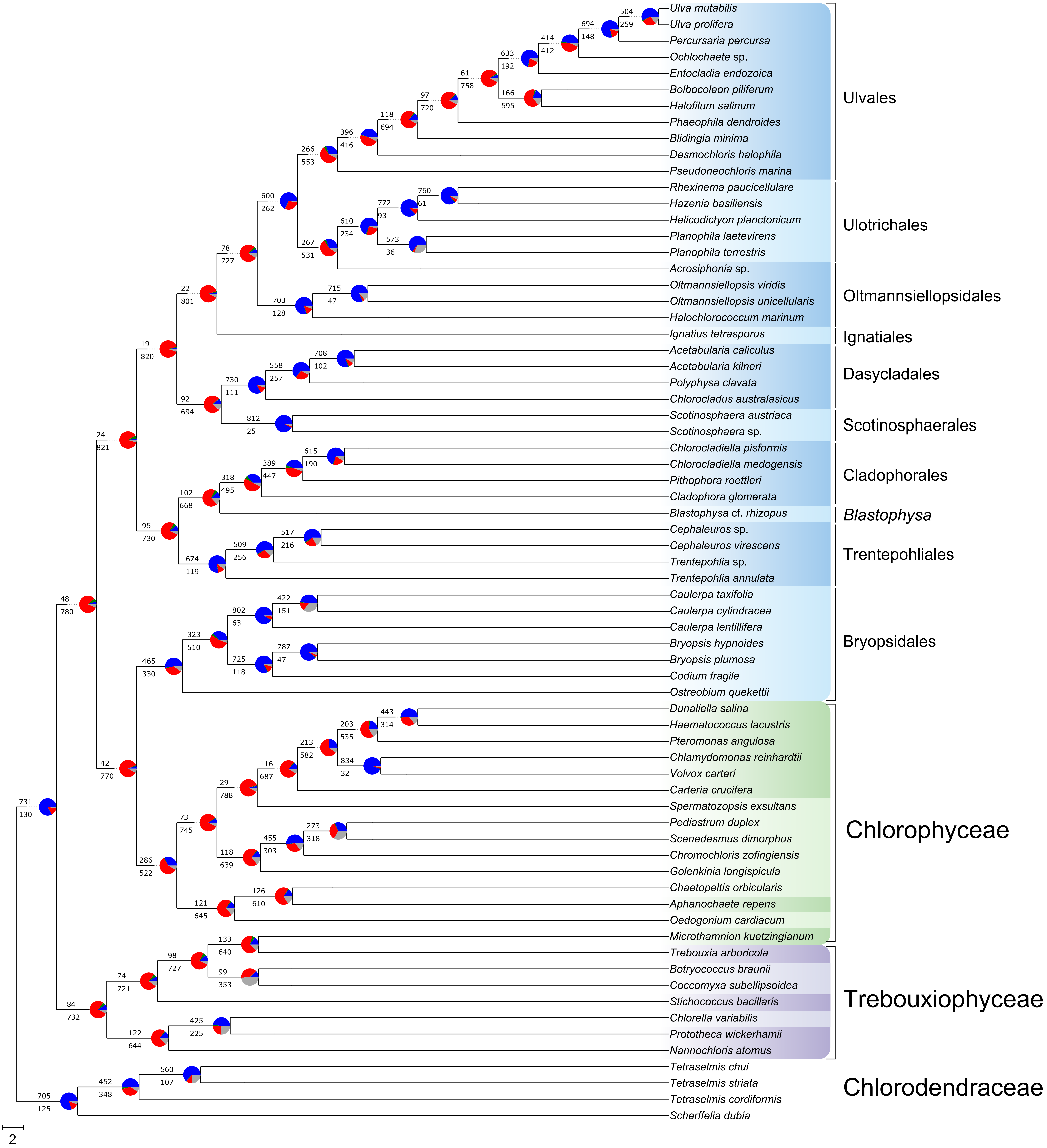

Supplementary Figure 4 The coalescence-based species tree of 884 nuclear genes with summary of gene tree concordance and conflict. Numbers above or below branches indicate the number of gene trees concordant or conflicting with that node in the species tree. Pie chart color coding: blue, fraction of gene trees that are concordant with the species tree; green, fraction of gene trees supporting the second most common conflicting topology; red, fraction of gene trees supporting all other alternative conflicting partitions; gray, fraction of gene trees with <50% bootstrap support at that node.

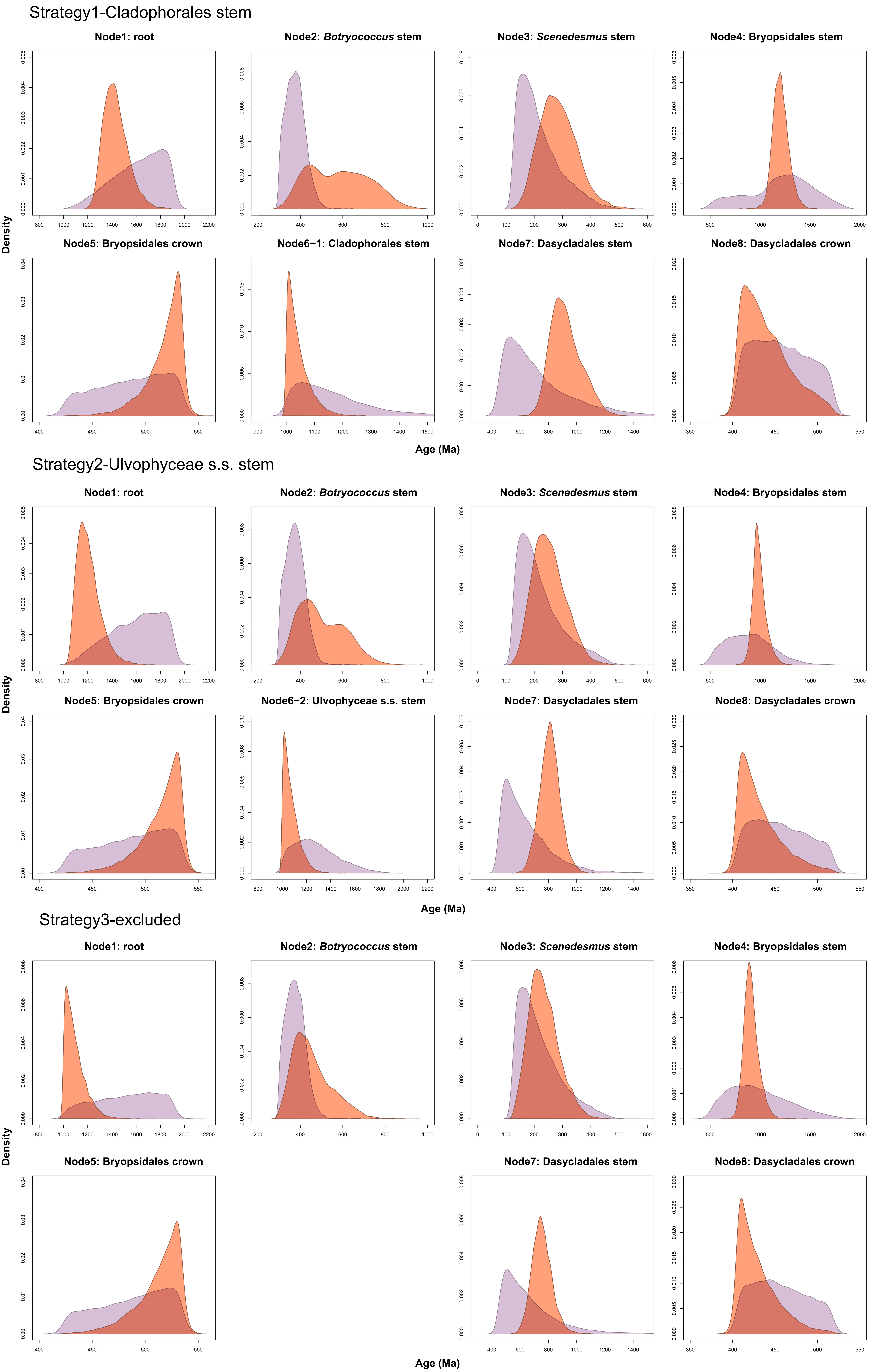

Supplementary Figure 5 Comparison of the probability density distributions of the effective prior (purple) and posterior (orange) on calibration nodes. The horizontal axis represents time (Ma). The source data are provided in the Source Data file.

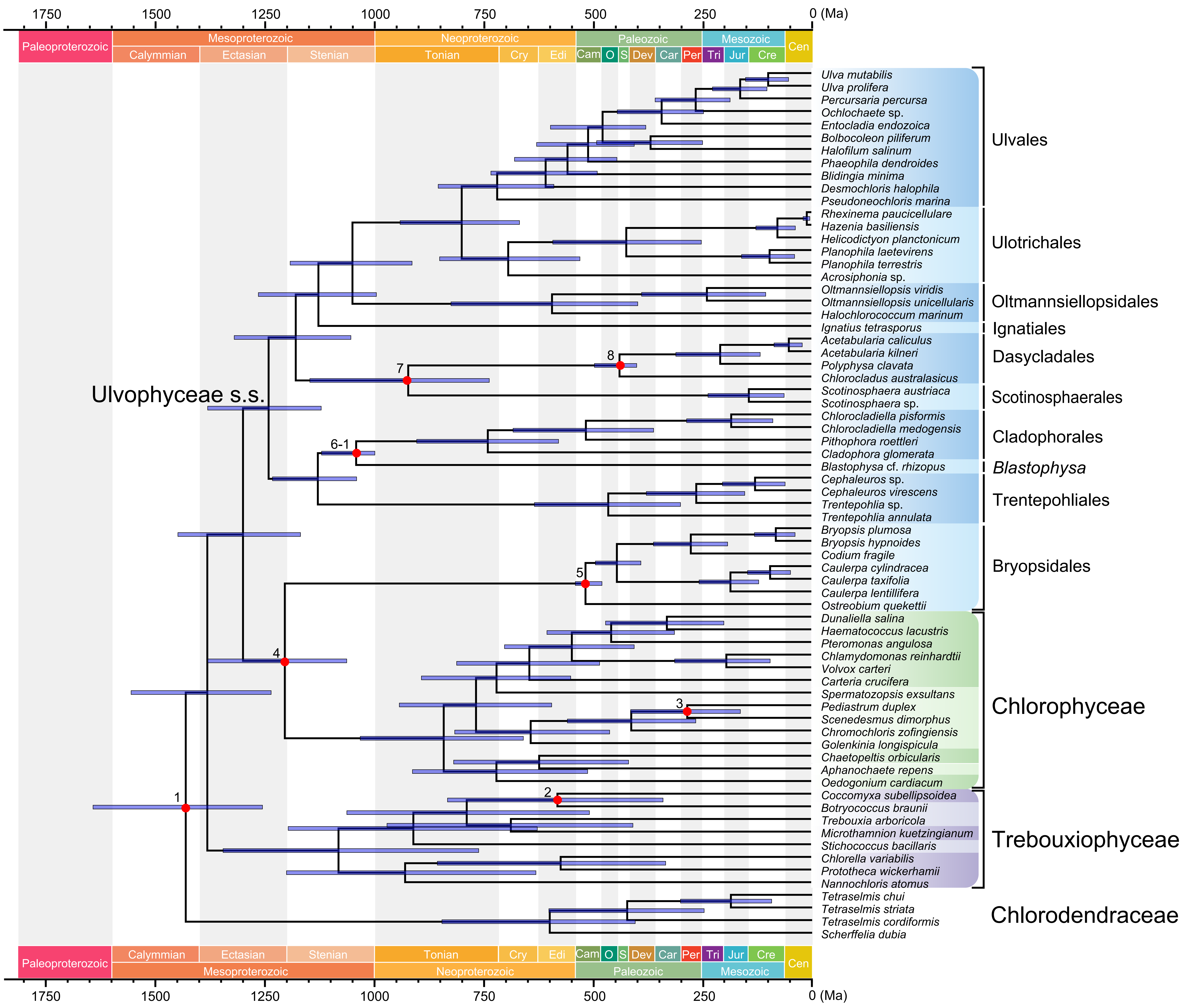

Supplementary Figure 6 The calibrated time tree of Ulvophyceae in strategy 1. Each node represents the mean posterior time, and their 95% credibility intervals are represented by the horizontal bars. The calibration nodes are represented by red dots.

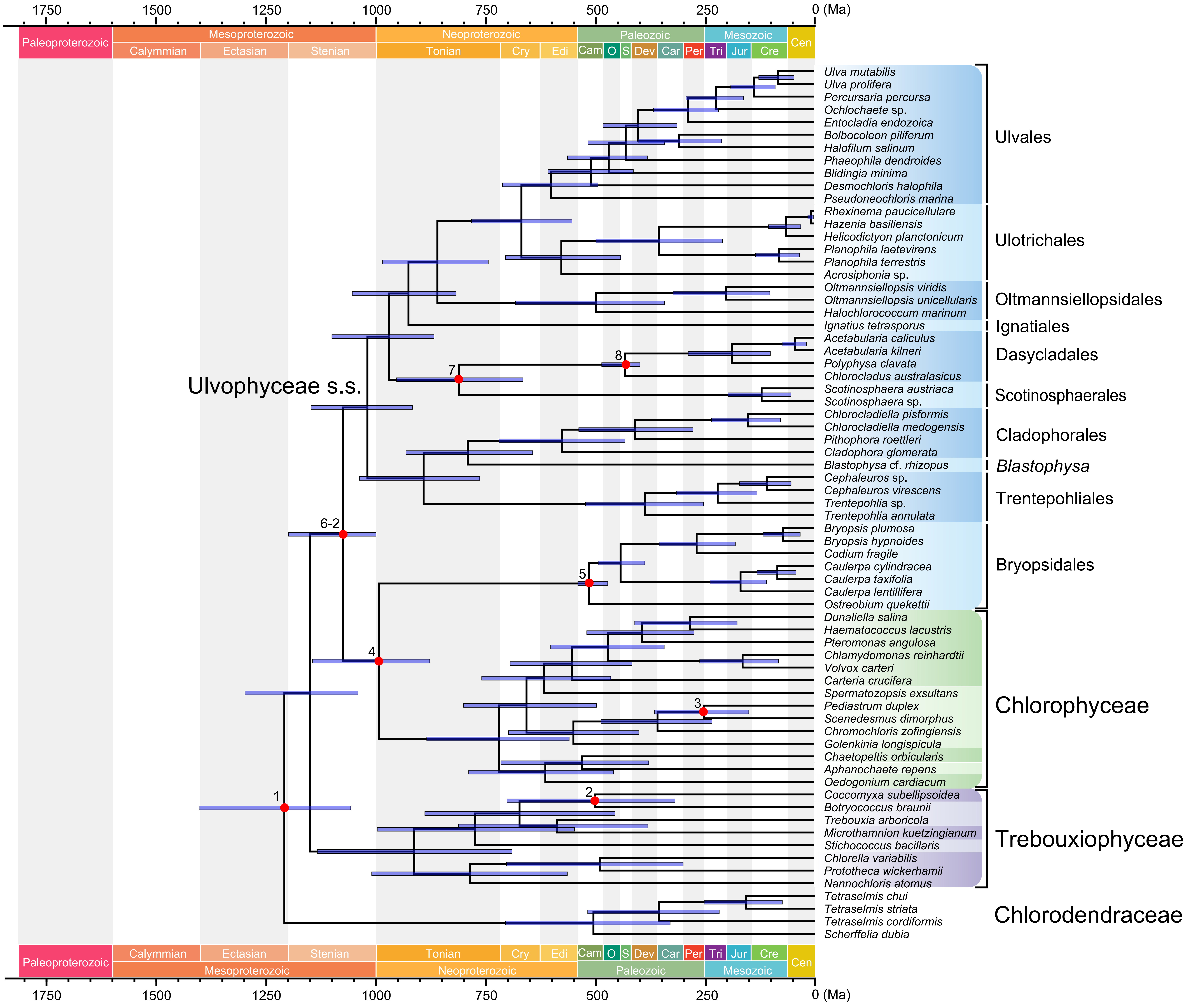

Supplementary Figure 7 The calibrated time tree of Ulvophyceae in strategy 2. Each node represents the mean posterior time, and their 95% credibility intervals are represented by the horizontal bars. The calibration nodes are represented by red dots.

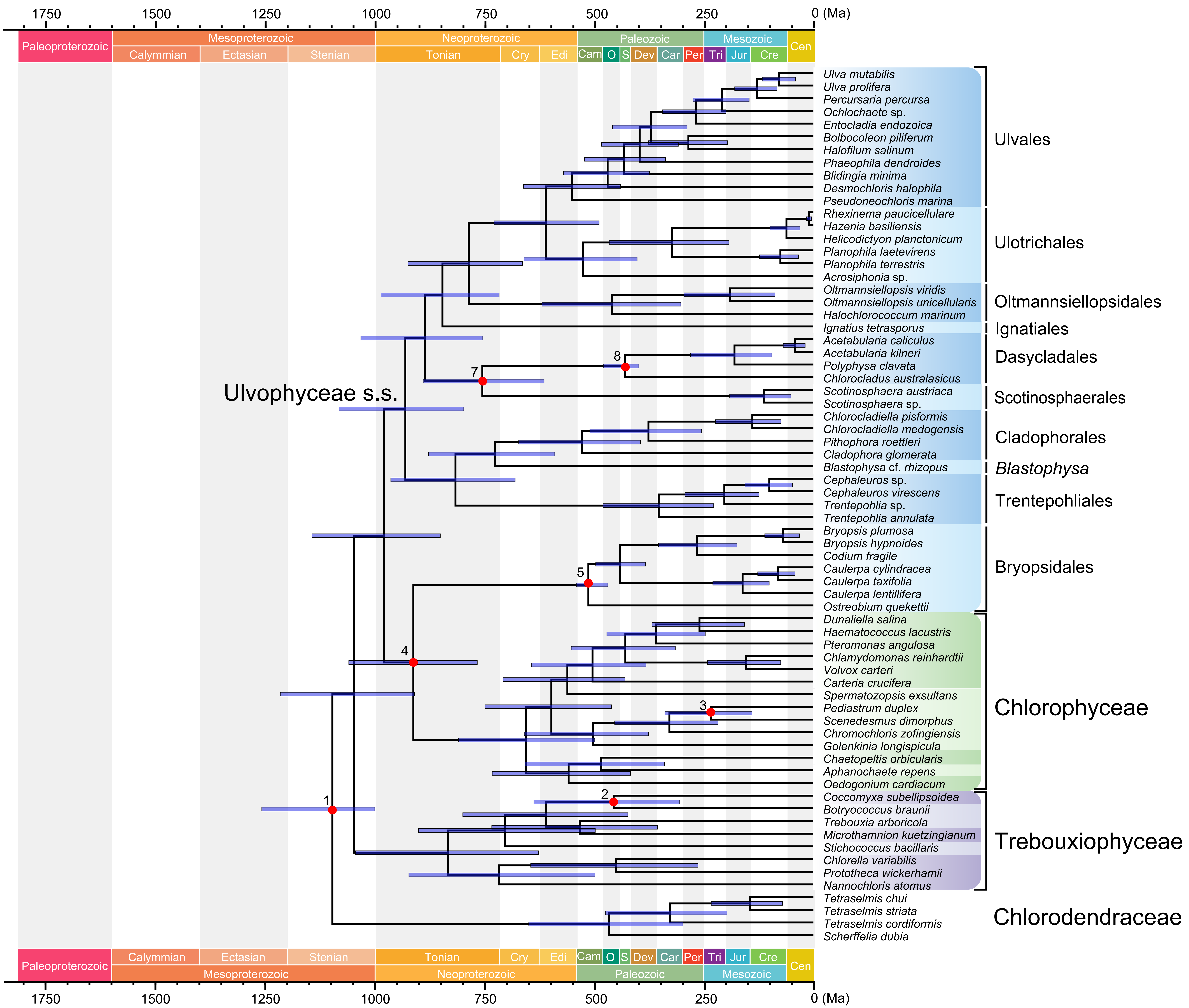

Supplementary Figure 8 The calibrated time tree of Ulvophyceae in strategy 3. Each node represents the mean posterior time, and their 95% credibility intervals are represented by the horizontal bars. The calibration nodes are represented by red dots.

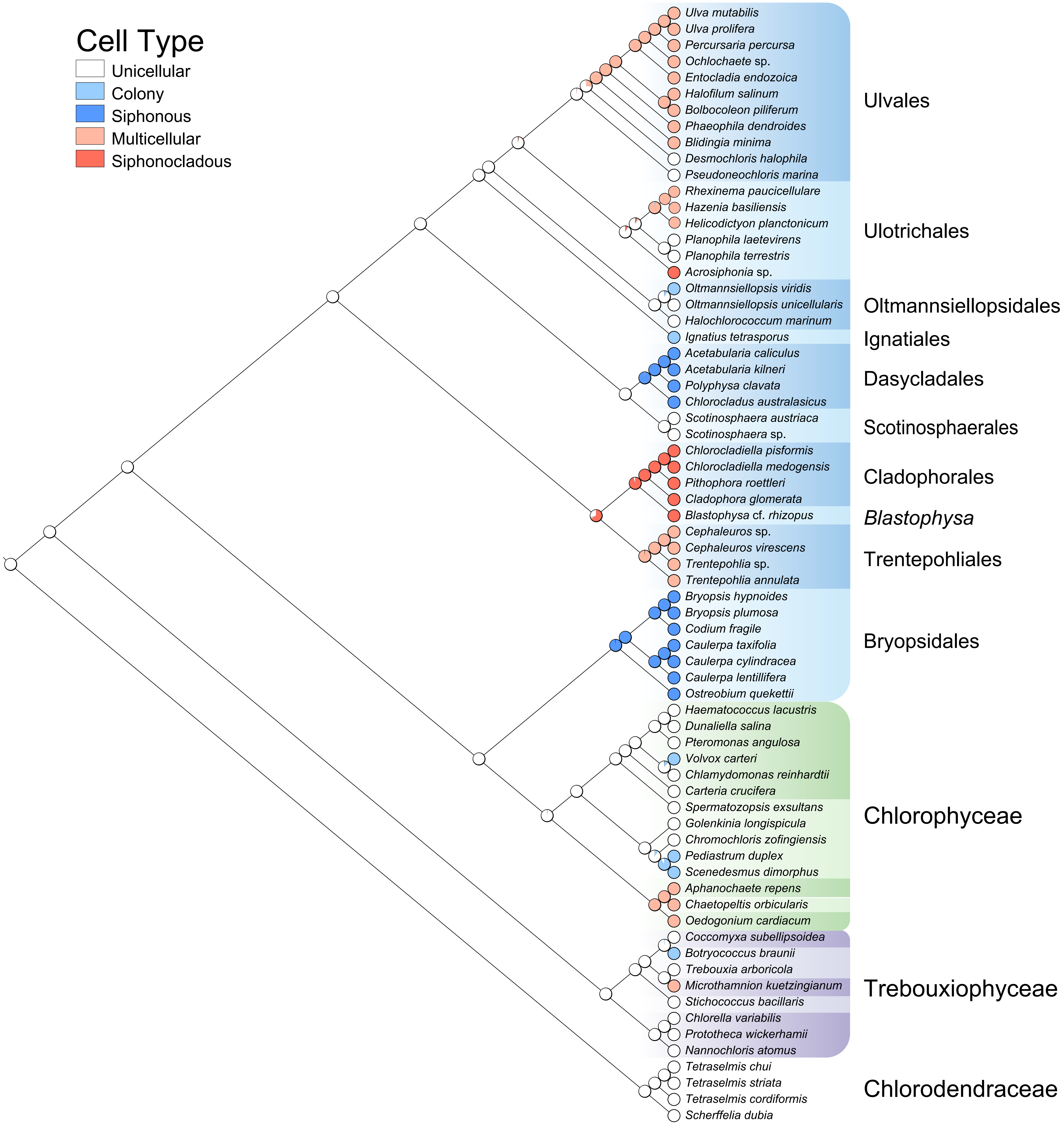

Supplementary Figure 9 Ancestral character estimation of cytomorphological traits based on maximum-likelihood method, and plotted on the tree in Supplementary Figure10.

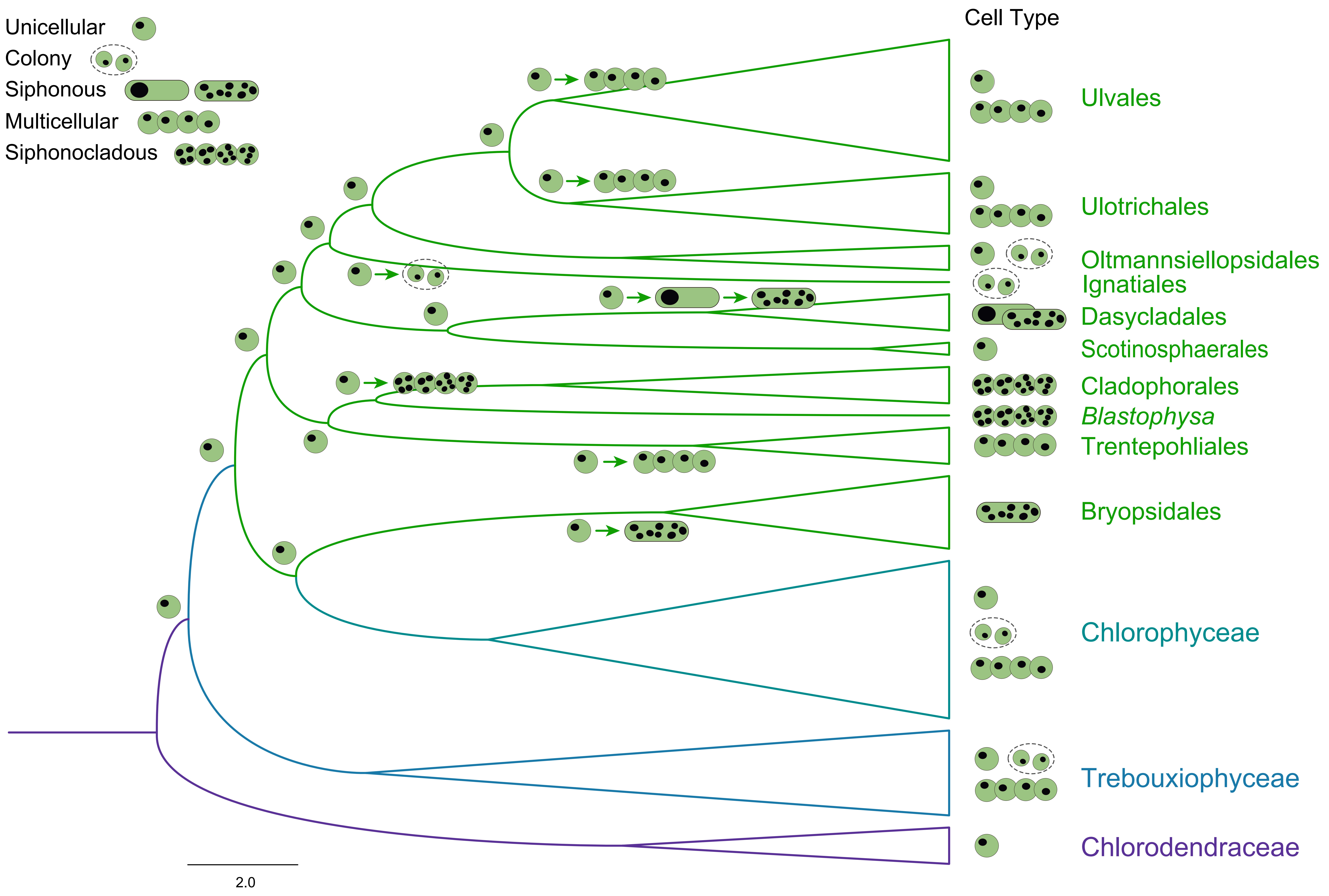

Supplementary Figure 10 The hypothesis about the evolution of multicellularity in the Ulvophyceae. The tree topology is based on the coalescence-based species tree.

Supplementary Note 1: Phylogenetic relationships and evolution of cyto-morphological and ultrastructural features

Traditional views on green alga relationships were largely based on the concept that evolution follows trends from simple to more complex morphological forms. In this view the morphologically complex green seaweeds were derived from simple unicellular and filamentous green microalgae[1]. Since the 1970s, a large amount of new data was gathered from life cycle studies, as well as from transmission electron microscopic investigations of green algal cells. In particular the fine structures of the flagellar apparatus, and the processes of mitosis and cytokinesis were studied and compared between green algal groups[2-6]. These ultrastructural features were assumed to more accurately reflect phylogenetic relationships because they are involved in fundamental eukaryotic processes of cell division and motility, and would thus be more conserved and less prone to convergent evolution than gross morphological characters. Since the 1990's, molecular phylogenetic data provided a new framework for reconstructing phylogenetic relationships, and increasing taxon and gene sampling have resulted in better supported and more reliable phylogenetic hypotheses[7-10]. The phylogeny in the current study allows testing ultrastructure-based hypotheses of green algal relationships, and putting forward some scenarios for the evolution of cyto-morphological and ultrastructural features.

Cytomorphological evolution

The present phylogeny confirms recent assumptions that macroscopic growth evolved from unicellular green algae through different mechanisms in the different lineages of the Ulvophyceae[8,11]. Contrary to some previous phylogenetic studies, which indicated a sister relationship between Bryopsidales and Dasycladales[11,12], siphonous thallus architecture likely evolved independently in the two orders. Likewise, multicellularity with coupled mitosis and cytokinesis likely evolved independently in the Ulvales-Ulotrichales and Trentepohliales, while there was only one transition from a unicellular ancestor to a siphonocladous thallus organization with uncoupled mitosis and cytokinesis in the Cladophorales and Blastophysa. The sister relationship of the Cladophorales-Blastophysa clade (mainly occurring in marine habitats) and the Trentepohliales (which are strictly terrestrial) may suggest that their common ancestor was multicellular. However, the mechanisms of cytokinesis are fundamentally different in these two clades. In the Trentepohliales, cell division is mediated by a phragmoplast, resulting in plasmodesmata, similar to what is found in the later diverging charophytic green algae[13-15]. In contrast, cell division in the Cladophorales takes place by ingrowth of a diaphragm-like cross wall, or by specialized modes of cell division that are unique in this order[7,16,17] (Supplementary Table 1). Given these fundamental cytological differences, it may be more probable that

multicellularity evolved independently from a unicellular ancestor in the Trentepohliales and the Cladophorales, possibly in response to different environmental pressures in terrestrial and marine habitats.

Evolution of the flagellar root system

The phylogenetic position of the Bryopsidales, sister to the Chlorophyceae, is unexpected from a cyto-morphological as well as ultrastructural view-point. The Bryopsidales share a counter-clockwise (CCW) orientation of basal bodies in the flagellar root system with overlapping basal bodies with most other orders of the Ulvophyceae, while the Chlorophyceae are characterized by a clock-wise (CW) or directly opposite (DO) orientation of basal bodies with non-overlapping basal bodies (Supplementary Table 1). The fact that the Trebouxiophyceae, Chlorodendrophyceae and Pedinophyceae also have a CCW flagellar root system, confirms earlier assumptions that a CCW orientation of the flagellar basal bodies is ancestral in the core Chlorophyta, and evolved to a DO and CW orientation in the Chlorophyceae[7].

Various relationships between orders have been proposed based on detailed features of the flagellar root system. For example, a close relationship between the Dasycladales, Cladophorales was proposed based on similar striations on the distal fibers connecting the basal bodies and lack terminal caps on the anterior surface of the basal bodies[5,18], while other authors suggested that the Dasycladales possesses ultrastructural features that are intermediate between the Chlorophyceae and the Ulvophyceae[19]. Instead, our results indicate a relationship between the Cladophorales and Trentepohliales, characterized by some unusual ultrastructure characters (Supplementary Table 1), indicating that the aforementioned flagellar features are less conserved than previously assumed. Although the ultrastructural details of the flagellar root system have been extensively studied in the main clades of the Ulvophyceae, the smaller clades, including the Oltmannsiellopsidales, Ignatiales and Scotinosphaerales remain largely understudied, hampering a complete picture of the evolution of flagellar ultrastructure in the group.

Modes of mitosis, cytokinesis, and other characters

Our phylogenetic results are broadly consistent with modes of mitosis and cytokinesis, and earlier hypothesis on the evolution of these traits[6,20,21]. A sister relationship between the Bryopsidales and Chlorophyceae would imply that a persistent telophase spindle (Supplementary Table 1) has evolved independently in the Bryopsidales and Ulvophyceae s.s., or alternatively that a persistent telophase spindle represents the ancestral state of the Ulvophyceae and Chlorophyceae. The fact that the Chlorophyceae, Trebouxiophyceae, and Chlorodendrophyceae

all have a non-persistent spindle support that this character state is ancestral, although the persistent spindle in the Pedinophyceae indicates that multiple evolutionary changes likely occurred.

Other cytological features, such as chloroplast morphology, and life cycle features, are variable between as well as within the main ulvophycean clades (Supplementary Table 1), and as a result they appear to be less phylogenetic informative at higher taxonomic levels. Cell wall composition is potentially informative at higher taxonomic levels[22-24], but detailed information is lacking for several of the smaller ulvophycean clades (Supplementary Table 1).

Supplementary Table 1: Ecological, morphological, cytological, ultrastructural, and life cycle features of the main clades in the Ulvophyceae and core Chlorophyta in regard to the phylogenetic relationship inferred in this study (Pedinophyceae are included for completeness). n.a.: data not available.

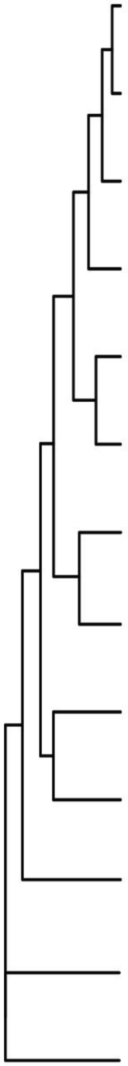

Clade and estimated species diversity [1]	Habitat [2]	Thallus architecture	Cytological organisation of vegetative cells	Orientation of basal bodies (bb) in the flagellar root system [9]	Cytokinesis	Mitosis	Chloroplast(s) in vegetative cells	Zoids	Cell wall composition [3]	Life cycle
**Ulvales** (250 species) [4]	Benthic, marine/brackish, freshwater/terrestrial	Macro- or microscopic multicellular filamentous, tubular or blade-like (some unicellular) [4]	Uninucleate	CCW, bb overlapping, perpendicular to axis of forward motion, rhizoplast present or absent	Furrowing, phycoplast absent	Closed, persistent telophase spindle	Single chloroplast, one to several pyrenoids	With 2 or 4 flagella, thin cell wall, without body scales	Irregularly arranged microfibrils embedded in an amorphous matrix of polysaccharides	Diplohaplontic iso- or heteromorphic, or asexual reproduction
**Ulotrichales** (250 species)	Benthic, marine/brackish, freshwater/terrestrial	Macroscopic multicellular or microscopic uni- or multicellular (coccoid, sarcinoid, filamentous or blade-like)	Uninucleate (sometimes multinucleate [4])	CCW, bb overlapping, perpendicular to axis of forward motion (sometimes V-shaped configuration), rhizoplast present or absent	Furrowing, phycoplast absent	Closed, persistent telophase spindle.	Single parietal, sometimes reticulate, one to several pyrenoids	With 2 or 4 flagella, thin cell wall, some with diamond-shaped scales	Irregularly arranged microfibrils embedded in an amorphous matrix of polysaccharides	Diplohaplontic iso- or heteromorphic, often with Codiolum phase, or asexual reproduction
**Oltmannsiellopsidales** (7 species)	Planktonic or benthic, marine, brackish, freshwater	Microscopic unicellular or colonial flagellates, coccoid cells or sarcinoid forms.	Uninucleate	CCW, bb overlapping, V-shaped configuration, rhizoplast not reported [10]	Furrowing, phycoplast not reported	unknown	Single cup-shaped with stigma and pyrenoid	With 4 flagella, cell wall thin or absent, with or without scales	unknown	Asexual reproduction
**Ignatiales** (4 species)	Benthic, freshwater, terrestrial	Microscopic unicellular, coccoid or sarcinoid	Uninucleate	CCW, bb non-overlapping, perpendicular to axis of forward motion, rhizoplast present [12]	Furrowing, phycoplast not reported	unknown	Single parietal, perforated, with stigma and one to several pyrenoids	With 4 flagella, cell wall thin or absent, scales absent	unknown	Asexual reproduction by quadriflagellate zoospores
**Dasycladales** (50 species)	Benthic marine	Macroscopic, siphonous, central axis with whorls of branches, often calcified	Uni- or multinucleate, cytoplasmic streaming [6]	CCW, bb overlapping, perpendicular to axis of forward motion, rhizoplast present or absent	n.a.	Closed, acentric, persistent telophase spindle	Numerous, ellipsoidal or fusiform, often with grains of reserve polysaccharide, pyrenoids absent	With 2 flagella, without cell wall or scales	Fibrillar structural fraction mannan-rich, cellulose in walls of gametangial cysts	Unique life cycle, interpreted as haplontic
**Scotinosphaerales** (9 species)	Benthic freshwater, terrestrial	Microscopic, unicellular, large coccoid cells	Uninucleate	CCW, bb overlapping, rhizoplast not reported	Furrowing, phycoplast absent	unknown	Single, with radiating and anastomosing lobes, two or more pyrenoids	Biflagellate, without cell wall or scales	unknown	Asexual reproduction by zoospores or autospores
**Trentepohliales** (105 species)	Terrestrial	Macroscopic, multicellular, filamentous or pseudoparenchymatous	Uninucleate	CCW, bb overlapping, perpendicular to axis of forward motion; with multilayered structure	Cell-plate formation with phragmoplast. Plasmodesmata	Open, persistent telophase spindle	Single, parietal, variable morphology, pyrenoids absent	With 2 or 4 flagella, compressed, cell wall and scales presumably absent	Cellulose, some species with pectic caps or sporopollenin	Diplohaplontic iso- or heteromorphic, or asexual reproduction
**Cladophorales** (400 species)	Benthic marine, brackish, freshwater	Macroscopic, multicellular (siphonocladous), filamentous or pseudoparenchymatous	Multinucleate, no cytoplasmic streaming [5, 7]	CCW, bb overlapping, perpendicular to axis of forward motion, rhizoplast absent	Furrowing or specialized modes of cell division. Phycoplast absent.	Closed, persistent telophase spindle. Mitosis uncoupled from cytokinesis	Numerous, with pyrenoid, forming a parietal network or continuous layer	With 2 or 4 flagella, lacking cell wall or scales but with dense tomentose layer [11]	Cellulose I with parallel microfibrils in numerous lamellae	Diplohaplontic and isomorphic, or reproduction asexual
**Bryopsidales** (520 species)	Benthic marine (one freshwater genus)	Macroscopic, acellular (siphonous), simple branched siphons to complex thalli	Multinucleate, cytoplasmic streaming [8]	CCW, bb overlapping, perpendicular to axis of forward motion, rhizoplast absent	n.a.	Closed, persistent telophase spindle. Mitosis asynchronously	Numerous, ellipsoidal or fusiform, with or without pyrenoid. Homo- or heteroplastidic.	With 2 or 4 flagella (or stephanokont), with or without cell wall, lacking scales	Mannan, xylan and glucan (cellulose)	Haplontic or diplohaplontic iso- or heteromorphic
**Chlorophyceae** (2300 species)	Freshwater (some terrestrial and marine)	Microscopic unicellular flagellates, coccoid cells, and multicellular branched filaments	Uninucleate	CW or DO, bb not overlapping, perpendicular to axis of forward motion or V-shaped, rhizoplast absent	Furrowing or cell plate formation mediated by phycoplast	Closed, non-persistent telophase spindle	Variable, ranging from cup-shaped to reticulate, pyrenoids present or absent	With two flagella (or stephanokont), scales present or absent	Glycoprotein envelope (flagellates), or polysaccharide walls (sometimes cellulose)	Haplontic with hypnozygote stage
**Trebouxiophyceae** (550 species)	Freshwater/terrestrial (some marine)	Microscopic unicellular coccoid, colonies or multicellular filaments or blades (macroscopic)	Uninucleate	CCW, bb overlapping, perpendicular to axis of forward motion, rhizoplast present	Furrowing, phycoplast present	Semi-closed, non-persistent telophase spindle	Variable, including parietal, stellate and netlike forms, pyrenoids present or absent.	With two flagella, compressed, scales absent	Cellulose, algaenans, and galactofuranan	Asexual reproduction by auto- or zoospores. Some species diplohaplontic heteromorphic
**Chlorodendrophyceae** (43 species)	Planktonic, marine, inland saline, brackish, (some freshwater)	Unicellular flagellates (sometimes forming stalked sessile colonies)	Uninucleate	CCW, bb not overlapping, parallel to axis of forward motion, rhizoplast present	Furrowing, phycoplast present	Closed, non-persistent telophase spindle	One or two per cell, with or without stigma and pyrenoid	With 4 flagella with hair-like scales, cells with theca formed by minute organic scales.	Fused scales, proteins and keto-sugars	Reproduction asexual
**Pedinophyceae** (22 species)	Planktonic, marine, brackish, freshwater, terrestrial	Unicellular flagellates	Uninucleate	CCW, bb with antiparallel orientation, rhizoplast present	Furrowing, phycoplast absent	Closed mitosis, persistent telophase spindle	Lateral chloroplast with stigma and pyrenoid	With 1 flagellum, asymmetrical, often compressed, cell wall absent, rarely covered by scales or a theca	Cell wall absent	Reproduction asexual

[1] Species diversity estimates based on ref. [25].
[2] Based on refs. [7,20,26-28].
[3] Based on refs. [20,23].
[4] Including the Chlorocystidales and Sykidiales, both including unicellular, coccoid species[28,29].
[5] Multinucleate cells without cytoplasmic streaming occur in the Ulotrichales (e.g., *Urospora* and *Acrosiphonia*) and Cladophorales. In contrast to the situation in the Cladophorales, species in the Ulotrichales synchronize mitosis and cytokinesis in vegetative cells of mature filaments[11,30].
[6] Siphons with large central vacuole surrounded by a thin layer of cytoplasm. Cytoplasmic streaming. Uninucleate (macronucleus) or multinucleate.
[7] Vegetative cells multinucleate with central vacuole surrounded by a thin layer of cytoplasm containing numerous chloroplasts and nuclei with fixed positions (no cytoplasmic streaming).
[8] Siphons with large central vacuole, surrounded by a thin layer of cytoplasm containing many plastids and nuclei. Cytoplasm exhibiting streaming.
[9] All species of the core Chlorophyta with flagellar stages have a flagellar apparatus with a symmetrical cruciate root system wherein rootlets of variable ('X') numbers of microtubules alternate with rootlets composed of two microtubules to form a "X-2-X-2" arrangement. The orientation of the flagellar roots has served as an important character for defining the main groups of Chlorophyta. When viewed from the anterior (flagellar) end of the cell, the flagellar basal bodies and rootlets can have a perfect cruciate pattern with basal bodies directly opposed (DO) or they are offset in a counter-clockwise (CCW) or clockwise (CW) position[5-7,20,27,31-33].
[10] Two internal basal bodies have a counterclockwise orientation, crossing each other at 45-90 degrees, while one external body is parallel to each internal basal body. This arrangement results in a characteristic flagellar orientation: a pair of flagella emerge on each side of the transverse axis of the colony[34].
[11] Based on ref. [35].
[12] The upper basal bodies are oriented CCW and non-overlapping; the lower basal bodies are DO or slightly CW.

Supplementary References

1 Fritsch, F. E. *The Structure and Reproduction of the Algae, Vol. I.,* (Cambridge University Press, 1935).

2 Picket-Heaps, J. D. & Marchant, H. J. The phylogeny of the green algae: A new proposal. *Cytobios* **6**, 255-264 (1972).

3 Mattox, K. R. & Stewart, K. D. in *Systematics of the green algae* (eds D. E. G. Irvine & D. M. John) 29-72 (Academic Press, 1984).

4 Melkonian, M. in *Systematics of the green algae* (eds D. E. G. Irvine & D. M. John) 73-120 (Academic Press, 1984).

5 O'Kelly, C. J. & Floyd, G. L. Flagellar apparatus absolute orientations and the phylogeny of the green algae. *Biosystems* **16**, 227-251 (1984).

6 van den Hoek, C., Stam, W. T. & Olsen, J. L. The emergence of a new chlorophytan system, and Dr. Kornmann's contribution thereto. *Helgol. Meeresunters.***42**, 339-383 (1988).

7 Leliaert, F. et al. Phylogeny and molecular evolution of the green algae. *Crit. Rev. Plant Sci.* **31**, 1-46, doi:10.1080/07352689.2011.615705 (2012).

8 Del Cortona, A. et al. Neoproterozoic origin and multiple transitions to macroscopic growth in green seaweeds. *Proc. Natl Acad. Sci. U.S.A.* **117**, 2551–2559, doi:10.1101/668475 (2020).

9 Gulbrandsen, Ø. S., Andresen, I. J., Krabberød, A. K., Bråte, J. & Shalchian-Tabrizi, K. Phylogenomic analysis restructures the Ulvophyceae. *J. Phycol*. (2021).

10 Li, X. et al. Large phylogenomic datasets reveal deep relationships and trait evolution in chlorophyte green algae. *Genome Biol. Evol.*, doi:10.1093/gbe/evab101 (2021).

11 Cocquyt, E., Verbruggen, H., Leliaert, F. & De Clerck, O. Evolution and cytological diversification of the green seaweeds (Ulvophyceae). *Mol. Biol. Evol*. **27**, 2052-2061, doi:10.1093/molbev/msq091 (2010).

12 Fučíková, K. et al. New phylogenetic hypotheses for the core Chlorophyta based on chloroplast sequence data. *Front. Ecol. Evol*. **2**, 63, doi:10.3389/fevo.2014.00063 (2014).

13 López-Bautista, J. M., Waters, D. A. & Chapman, R. L. The Trentepohliales revisited. *Constancea* **83** (2002).

14 Chapman, R. L., Borkhsenious, O. L., Brown, R. C., Henk, M. C. & Waters, D. A. Phragmoplast-mediated cytokinesis in Trentepohlia: results of TEM and immunofluorescence cytochemistry. *Int. J.Syst. Evol. Microbiol*. **51**, 759-765 (2001).

15 Becker, B. & Marin, B. Streptophyte algae and the origin of embryophytes. *Ann. Bot.* **103**, 999-1004, doi:10.1093/aob/mcp044 (2009).

16 Okuda, K., Mine, I., Morinaga, T. & Kuwaki, N. Cytomorphogenesis in coenocytic green algae. V. Segregative cell division and cortical microtubules in Dictyosphaeria cavernosa (Siphonocladales, Chlorophyceae). *Phycol. Res.* **45**, 189-196 (1997).

17 Okuda, K. et al. Segregative cell division and the cytoskeleton in two species of the genus Struvea (Cladophorales, Ulvophyceae, Chlorophyta). *Phycol. Res.* **64**, 219-229, doi:10.1111/pre.12139 (2016).

18 Chappell, D. F., Okelly, C. J. & Floyd, G. L. Flagellar apparatus of the biflagellate zoospores of the enigmatic marine green-alga *Blastophysa rhizopus*. *J. Phycol*. **27**, 423-428 (1991).

19 Herth, W., Heck, B. & Koop, H. The flagellar root system in the gamete of Acetabularia mediterranea. *Protoplasma* **109**, 257-269 (1981).

20 van den Hoek, C., Mann, D. G. & Jahns, H. M. *Algae: an introduction to phycology*. (Cambridge University Press, 1995).

21 Zechman, F. W., Theriot, E. C., Zimmer, E. A. & Chapman, R. L. Phylogeny of the Ulvophyceae (Chlorophyta): cladistic analysis of nuclear-encoded rRNA sequence data. *J. Phycol.* **26**, 700-710, doi:10.1111/j.0022-3646.1990.00700.x (1990).

22 Ciancia, M., Fernández, P. V. & Leliaert, F. Diversity of Sulfated Polysaccharides From Cell Walls of Coenocytic Green Algae and Their Structural Relationships in View of Green Algal Evolution. *Front. Plant Sci*. **11**, doi:10.3389/fpls.2020.554585 (2020).

23 Domozych, D. et al. The cell walls of green algae: a journey through evolution and diversity. *Front. Plant Sci*. **3**, doi:10.3389/fpls.2012.00082 (2012).

24 Domozych, D. S. Algal cell walls. *e LS* (2019).

25 Guiry, M. D. How many species of algae are there? *J. Phycol*. **48**, 1057-1063, doi:10.1111/j.1529-8817.2012.01222.x (2012).

26 Brodie, J., Maggs, C. A. & John, D. M. *Green seaweeds of Britain and Ireland*. **242** (British Phycological Society, 2007).

27 Graham, L. E., Graham, J. M., Wilcox, L. W. & Cook, M. E. *Algae (Third Edition)*. (LJLM Press, 2016).

28 Škaloud, P., Rindi, F., Boedeker, C. & Leliaert, F. *Freshwater Flora of Central Europe, Vol 13: Chlorophyta: Ulvophyceae*. Vol. 13 1-288 (Springer Spektrum, 2018).

29 Darienko, T., Rad-Menéndez, C., Campbell, C. N. & Pröschold, T. Molecular Phylogeny of Unicellular Marine Coccoid Green Algae Revealed New Insights into the Systematics of the Ulvophyceae (Chlorophyta). *Microorganisms* **9**, 1586 (2021).

30 Lokhorst, G. M. & Star, W. Fine-structure of mitosis and cytokinesis in Urospora (Acrosiphoniales, Chlorophyta). *Protoplasma* **117**, 142-153 (1983).

31 Nakayama, T., Watanabe, S. & Inouye, I. Phylogeny of wall-less green flagellates inferred from 18S rDNA sequence data. *Phycol. Res*. **44**, 151-161 (1996).

32 Watanabe, S. & Nakayama, T. Ultrastructure and phylogenetic relationships of the unicellular green algae Ignatius tetrasporus and Pseudocharacium americanum (Chlorophyta). *Phycol. Res.* **55**, 1-16, doi:10.1111/j.1440-1835.2006.00439.x (2007).

33 Škaloud, P., Kalina, T., Nemjová, K., De Clerck, O. & Leliaert, F. Morphology and phylogenetic position of the freshwater green microalgae Chlorochytrium (Chlorophyceae) and Scotinosphaera (Scotinosphaerales, ord. nov., Ulvophyceae). J. Phycol. 49, 115-129, doi:10.1111/jpy.12021 (2013).

34 Chihara, M., Inouye, I. & Takahata, N. Oltmannsiellopsis, a new genus of marine flagellate (Dunaliellaceae, Chlorophyceae). *Arch. Protistenk.* **132**, 313-324, doi:10.1016/S0003-9365(86)80026-4 (1986).

35 Bakker, M. E. & Lokhorst, G. M. The ultrastructure of the flagellar apparatus of the zoospore of Chaetomorpha melagonium (Web. & Mohr) Kützing (Chlorophyta). *Phycologia* **24**, 275-288, doi:10.2216/i0031-8884-24-3-275.1 (1985).
