# Supplementary material for: Phylotranscriptomic insights into a Mesoproterozoic–Neoproterozoic origin and early radiation of green seaweeds (Ulvophyceae)
Source: Nat Commun. 2022 Mar 22;13:1610. doi: 10.1038/s41467-022-29282-9 (PMC8941102; doi:10.1038/s41467-022-29282-9)
Supplement: Supplementary file 2 — Description of Additional Supplementary Files [file 41467_2022_29282_MOESM2_ESM.pdf]

Description of Additional Supplementary Files

Supplementary Data 1: Taxonomic information and sources for the species used in this study.
