# Supplementary material for: Phylotranscriptomic insights into a Mesoproterozoic–Neoproterozoic origin and early radiation of green seaweeds (Ulvophyceae)
Source: Nat Commun. 2022 Mar 22;13:1610. doi: 10.1038/s41467-022-29282-9 (PMC8941102; doi:10.1038/s41467-022-29282-9)
Supplement: Supplementary file 4 — Reporting Summary [file 41467_2022_29282_MOESM4_ESM.pdf]

nature portfolio

Corresponding author(s): Bojian Zhong

Last updated by author(s): Feb 15, 2022

Reporting Summary

Nature Portfolio wishes to improve the reproducibility of the work that we publish. This form provides structure for consistency and transparency in reporting. For further information on Nature Research policies, see Editorial Policies and the Editorial Policy Checklist.

Statistics

For all statistical analyses, confirm that the following items are present in the figure legend, table legend, main text, or Methods section.

n/a	Confirmed	
☐	☒	The exact sample size (*n*) for each experimental group/condition, given as a discrete number and unit of measurement
☒	☐	A statement on whether measurements were taken from distinct samples or whether the same sample was measured repeatedly
☒	☐	The statistical test(s) used AND whether they are one- or two-sided<br>*Only common tests should be described solely by name; describe more complex techniques in the Methods section.*
☒	☐	A description of all covariates tested
☒	☐	A description of any assumptions or corrections, such as tests of normality and adjustment for multiple comparisons
☐	☒	A full description of the statistical parameters including central tendency (e.g. means) or other basic estimates (e.g. regression coefficient) AND variation (e.g. standard deviation) or associated estimates of uncertainty (e.g. confidence intervals)
☒	☐	For null hypothesis testing, the test statistic (e.g. *F*, *t*, *r*) with confidence intervals, effect sizes, degrees of freedom and *P* value noted<br>*Give P values as exact values whenever suitable.*
☐	☒	For Bayesian analysis, information on the choice of priors and Markov chain Monte Carlo settings
☒	☐	For hierarchical and complex designs, identification of the appropriate level for tests and full reporting of outcomes
☐	☒	Estimates of effect sizes (e.g. Cohen's *d*, Pearson's *r*), indicating how they were calculated

Our web collection on statistics for biologists contains articles on many of the points above.

Software and code

Policy information about availability of computer code

Data collection	Softwares used for data collection include: Illumina Novaseq platform, Trinity v2.5.1, Corset.
Data analysis	Softwares used for data analysis include: OrthoMCL v2.0.9, Orthograph v0.6.3, MAFFT v7.310, Gblocks v0.91b, trimAl v1.2, IQ-TREE v1.6.12, , Newick Utilities v1.6, ASTRAL v5.7.3, PhyParts, PhyPartsPieCharts, ETE3, Phybase v1.5, PAML package v4.9j, SortaDate, Tracer v1.7.1, MCMCTreeR v1.1, R v4.0.2, L-INS-I algorithm, CODEML.

For manuscripts utilizing custom algorithms or software that are central to the research but not yet described in published literature, software must be made available to editors/reviewers. We strongly encourage code deposition in a community repository (e.g. GitHub). See the Nature Research guidelines for submitting code & software for further information.

Data

Policy information about availability of data

All manuscripts must include a data availability statement. This statement should provide the following information, where applicable:

- Accession codes, unique identifiers, or web links for publicly available datasets
- A description of any restrictions on data availability
- For clinical datasets or third party data, please ensure that the statement adheres to our policy

The raw Illumina data generated for this study are available through the Sequence Read Archive (SRA accession PRJNA726747). The alignment data and phylogenetic trees are available from Figshare: https://figshare.com/s/40126faad551cdfccf69.
Links to public databases mentioned in the manuscript are as follows: AlgaeBase: https://www.algaebase.org/, SRR7168057: https://www.ncbi.nlm.nih.gov/sra/?term=SRR7168057, 1kp: https://db.cngb.org/onekp/, SRR3524808: https://www.ncbi.nlm.nih.gov/sra/?term=SRR3524808, Phytozome v12: https://phytozome-next.jgi.doe.gov/

Field-specific reporting

Please select the one below that is the best fit for your research. If you are not sure, read the appropriate sections before making your selection.

☐ Life sciences ☐ Behavioural & social sciences ☒ Ecological, evolutionary & environmental sciences

Ecological, evolutionary & environmental sciences study design

All studies must disclose on these points even when the disclosure is negative.

Study description	Using more extensive nuclear gene dataset and coalescent- and concatenation-based approaches to infer: the phylogenetic relationships and the temporal framework of the diversification of the Ulvophyceae.
Research sample	Our research sample contained species of four classes in the core Chlorophyta: UTC(Ulvophyceae, Chlorophyceae and Trebouxiophyceae)+Chlorodendrophyceae. Our research focuses on the phylogenetic relationship and divergence time of Ulvophyceae. Therefore, we selected more Ulvophyceae sample, and a small number of Chlorophyceae and Trebouxiophyceae sample and Chlorodendrophyceae as outgroup.
Sampling strategy	The basic principle for determining the sample size is: by sequencing new strains and downloading public database data, all orders of Ulvophyceae are covered as much as possible, and each order has at least 1~2 species. Representative species were selected from Chlorophyceae and Trebouxiophyceae. Our main study is Ulvophyceae and the phylogenetic relationship of the major lineages of Ulvophyceae, so these sample sizes are sufficient.
Data collection	The publicly available data was downloaded from the public database by Zheng Hou using a computer. The culture and enrichment of 11 new strains were completed by Zheng Hou, and the subsequent transcriptome sequencing was performed using the Illumina Novaseq platform, at Novogene Bioinformatics Technology Co., Ltd (Beijing, China).
Timing and spatial scale	The public data collection period was 2018.09-2019.04 for eight months, and available genomic and transcriptomic data were downloaded from public databases. The 11 new strains data collection period was 2018.09-2020.01 for 17 months. They were cultivated in the laboratory and then transcriptome sequencing was performed using the Illumina Novaseq platform, at Novogene Bioinformatics Technology Co., Ltd (Beijing, China).
Data exclusions	No data were excluded.
Reproducibility	To ensure reproducibility of experimental results, we provide the raw Illumina data and the alignment data, and the software and parameters used for each analysis are described in detail in the Materials and Methods. We consider all experimental results to be reproducible.
Randomization	Bootstrap support for each node in gene trees and species trees was estimated by random resampling of sequence alignments.
Blinding	The study did not involve experimental manipulation. As such, blinding was not part of the research strategy.

Did the study involve field work? ☐ Yes ☒ No

Reporting for specific materials, systems and methods

We require information from authors about some types of materials, experimental systems and methods used in many studies. Here, indicate whether each material, system or method listed is relevant to your study. If you are not sure if a list item applies to your research, read the appropriate section before selecting a response.

Materials & experimental systems

n/a	Involved in the study
☒	☐ Antibodies
☒	☐ Eukaryotic cell lines
☒	☐ Palaeontology and archaeology
☒	☐ Animals and other organisms
☒	☐ Human research participants
☒	☐ Clinical data
☒	☐ Dual use research of concern

Methods

n/a	Involved in the study
☒	☐ ChIP-seq
☒	☐ Flow cytometry
☒	☐ MRI-based neuroimaging
